# Integrative Analysis of the Doxorubicin-Associated LncRNA–mRNA Network Identifies Chemoresistance-Associated lnc-TRDMT1-5 as a Biomarker of Breast Cancer Progression

**DOI:** 10.3389/fgene.2020.00566

**Published:** 2020-05-29

**Authors:** Qi Chen, Hui Yang, Xiaolan Zhu, Shangwan Xiong, Huamao Chi, Wenlin Xu

**Affiliations:** ^1^Department of Breast Diseases, Fourth Affiliated Hospital of Jiangsu University, Zhenjiang, China; ^2^School of Medicine, Jiangsu University, Zhenjiang, China; ^3^Central Laboratory, Fourth Affiliated Hospital of Jiangsu University, Zhenjiang, China

**Keywords:** breast cancer, GEO datasets, chemoresistance, lncRNA, prognosis

## Abstract

Increasing evidence has revealed close relationships between long non-coding RNAs (lncRNAs) and chemoresistance in multiple types of tumors; however, functional lncRNAs in breast cancer (BC) have not been completely identified. In this study, we aimed to identify novel lncRNAs that might play critical roles in doxorubicn resistance, which could reveal potential biomarkers of BC. Using a BC dataset (GSE81971), we identified 452 lncRNAs that were upregulated and 659 that were downregulated; furthermore, there were 1896 differentially expressed mRNAs, of which 1137 were upregulated and 758 were downregulated in MCF-7/ADR cells compared with the expression in MCF-7 cells. We constructed an lncRNA–mRNA network by integrating probe reannotation and regulatory interactions. To elucidate the key lncRNAs in BC, we further analyzed dysregulated lncRNA–mRNA crosstalk, and six candidate lncRNAs (lnc-TRDMT1-5, ZNF667-AS1, lnc-MPPE1-13, DSCAM-AS1:5, DSCAM-AS1:2, and lnc-CFI-3) were identified. Notably, the expression level of lnc-TRDMT1-5 was significantly upregulated in resistant cells compared with sensitive cells, and its levels were increased in BC tissues compared with adjacent tissues. Levels were positively associated with estrogen receptor (ER) and human epidermal growth factor receptor 2 (HER2) expression levels. High expression of lnc-TRDMT1-5 predicted poor prognosis in ER-positve and HER2-positive BC patients, especially in patients with chemoresistance. Bioinformatic and functional analysis revealed that lnc-TRDMT1-5 was involved in many crucial pathways in cancer, such as the PI3K/AKT and Wnt signaling pathways. Subcellular localization predicted that lnc-TRDMT1-5 was located in the cytoplasm, and the lncRNA–miRNA–mRNA network showed that lnc-TRDMT1-5 might serve as a regulator in BC. Here, our results demonstrated a dysregulated lncRNA–mRNA network that might provide new treatment strategies for chemoresistant BC, and the results identified a new lncRNA, lnc-TRDMT1-5, with oncogenic and prognostic functions in human BC.

## Introduction

Breast cancer (BC) is one of the most commonly diagnosed cancers among women (Siegel et al., 2017). Surgery and chemotherapy are the principal treatments for BC, but chemoresistance has become a major obstacle to achieving optimal prognosis in patients treated with systemic therapy (Runowicz et al., 2016). Studies have revealed that long non-coding RNAs (lncRNAs) are involved in multiple biological processes in the regulation of gene expression, including transcriptional, and posttranscriptional regulation, enabling them to play crucial roles in cancer metastasis and to increase chemoresistance (Xue et al., 2016) and rates of poor prognois (Tordonato et al., 2015). Although researchers have focused on the underlying chemoresistant mechanisms, the critical roles of lncRNAs in chemoresistant BC cells have not been completely determined. Therefore, it is important to study the functional lncRNAs related to chemoresistance and prognosis in BC.

LncRNAs are a diverse group of transcripts greater than 200 nucleotides in length, most of which do not encode proteins (Matsui and Corey, 2017). It is thought that the lncRNA–mRNA network provides a different approach to study the function of lncRNAs in cancer development (Liu et al., 2015; Schmitt and Chang, 2016). Doxorubicin (DOX), also known as adriamycin (ADR), is used for the chemotherapeutic treatment of various malignant diseases, including BC, ovarian cancer, and gastric cancer, and it is also used in combination with other chemotherapeutic agents (Pan et al., 2016). However, specific functional lncRNAs, as well as their underlying activity in DOX-resistant BC, remain to be further characterized.

Due to the large amount of microarray data, effectively screening functional lncRNAs is a challenge. Studies have shown close correlations among aberrantly expressed lncRNAs and mRNAs with regard to tumorigenesis, progression, and therapeutic potential (Tang et al., 2019). As lncRNAs interact with a variety of mRNAs, it is thought that lncRNA–mRNA networks provide important clues for in-depth exploration of lncRNA function (Zhang et al., 2018, 2020). Network-based methods can capture implicit feature information in the topology of lncRNA-related biological networks, offering a suitable method for acquiring more dependable results (Zhang et al., 2019).

Currently, specific lncRNAs typically have been putatively predicted via microarray or next-generation sequencing (NGS) analysis, after which they are experimentally validated in human cancer tissues or cells. These findings have provided mechanistic insights at the RNA level, which is widely used for biomarker-based prediction (Chen et al., 2017). Previous work based on the analysis of raw microarray data from the GSE53137 dataset has shown that upregulation of Linc00324 is positively correlated with advanced TNM stage (stage III), larger tumor size, and lymph node metastasis, as well as with poor prognosis, revealing that it could act as an oncogene in gastric cancer (Zou et al., 2018). Another lncRNA, Linc01638, functions to maintain mesenchymal features and the cancer stem cell-like status in cells by preventing SPOP-mediated c-Myc ubiquitination and degradation in triple-negative BC (TNBC; Luo et al., 2018). In addition, growth arrest-specific transcript 5 (GAS5), metastasis associated lung adenocarcinoma transcript 1 (MALAT1), and nuclear-enriched abundant transcript 1 (NEAT1) lncRNAs regulate BC cell invasion and proliferation and confer chemoresistance (Arshi et al., 2018). Taken together, these findings demonstrate that lncRNAs could be considered new biomarkers for cancer diagnosis and prognosis.

In this study, we examined genome-wide lncRNA expression profiles from the Gene Expression Omnibus (GEO) dataset containing three duplicated MCF-7/ADR (DOX-resistant) and MCF-7 (DOX-sensitive) BC cell lines. We identified differentially expressed mRNAs (DE-mRNAs) and lncRNAs (DE-lncRNAs), which were found to be functionally dysregulated in chemoresistant groups compared to sensitive groups. Based on bioinformatic and correlation analysis, six hub lncRNAs were selected to construct an lncRNA–mRNA-pathway network. Furthermore, we verified a novel lncRNA, lnc-TRDMT1-5, which was not only upregulated in BC tissue samples and resistant cells but also related to poor prognosis in estrogen receptor (ER)-positive, human epidermal growth factor receptor 2 (HER2) -positive, and chemoresistant BC patients. Thus, our study uncovered an lncRNA-mRNA regulatory network and identified lnc-TRDMT1-5 as a prognostic biomarker and therapeutic target for BC.

## Materials and Methods

### Cancer Tissue Samples and Cell Lines

A total of 20 pairs of BC and adjacent non-tumorous tissues were collected from patients who underwent surgical resection at the Fourth Affiliated Hospital of Jiangsu University (Jiangsu, China) from 2017 to 2018. All patients were diagnosed with BC by pathological identification, including invasive ductal carcinoma (IDC) and ductal carcinoma *in situ* (DCIS), and had no prior history of BC. All the samples were preserved in liquid nitrogen and then stored at −80°C.

The human BC cell line MCF-7/ADR (Shanghai Antique Biotechnology Company, China; Catalog No: BG006) was cultured in RPMI1640 medium (HyClone; Fcmacs Biotech Co., Ltd, Jiangsu, China), and MCF-7 cells (Stem Cell Bank, Chinese Academy of Sciences, Shanghai, China. Catalog No: TCHu-74) were cultured in MEM (HyClone; Fcmacs Biotech Co., Ltd., Jiangsu, China), both media contained 10% fetal bovine serum (FBS; HyClone; Fcmacs Biotech Co., Ltd, Jiangsu, China), 100 U/ml penicillin and 100 μg/ml streptomycin. The cells were incubated at 37°C in a 5% CO_2_ incubator. MCF-7/ADR cells were cultured with 1 μg/ml DOX in the medium.

### Microarray Analysis

The gene expression profile of GSE81971 was downloaded from GEO datasets,^1^ including MCF-7/ADR and MCF-7 cells. The gene expression platform was GPL19612. Samples in the dataset were analyzed with Agilent-062918 OE human lncRNA microarray V4.0 (probe name version). The gene profiles were compared with transcripts from NCBI RefSeq, NON-CODE v4, and GENCODE V18 using the BLAST program. The significant microarray analyses identified gene expression changes in MCF-7/ADR cells and MCF-7 cells (control) by calculating the significance level of the *P*-value and false discovery rate (FDR < 0) to screen for differentially expressed genes (DEGs), which were visualized using hierarchical clustering. The strict thresholds were set at fold change (| log_2_FC|) ≥ 2 and *P*-value < 0.05. Detailed Information on the different lncRNAs was exported from LNCipedia.^2^

### Functional Enrichment Analyses

To further delve into the enrichment analyses of the 1057 aberrantly expressed mRNAs described above, GO and KEGG analyses were performed using the Database for Annotation, Visualization and Integrated Discovery (DAVID)^3^ prior to analyzing the enrichment and functions of genes from three categories: biological process (BP), cellular component (CC) and molecular function (MF). A *P*-value < 0.05 was chosen as the cut-off criterion for GO functional and KEGG pathway enrichment analyses. Then, we selected mRNAs that overlapped in both GO and KEGG pathway enrichment analysis for further study.

### lncRNA–mRNA Coexpression Network

Most novel lncRNAs cannot be functionally annotated; thus, the functional annotation of mRNAs co-expressed with the lncRNAs was used to predict their function. Based on the expression level of DE-lncRNAs and DE-mRNAs, an lncRNA–mRNA coexpression analysis was conducted according to the normalized signal intensity of specific genes. According to the mRNA and lncRNA signal expression values, the correlation coefficient was used to fit the scale-free network relationship between the mRNA and lncRNA. An lncRNA-mRNA coexpression network was constructed to determine their association, the Pearson correlation coefficient (PCC) was calculated and the *R* value (Pearson’s *R*) was used to evaluate the correlation between lncRNAs and mRNAs. The data was screened and data for which the PCC was >0.99 were selected. The gene coexpression network was constructed using Cytoscape software version 3.1.1 (U.S. National Institute of General Medical Sciences, Washington, DC, United States). In the network, nodes represent genes. An edge between two nodes represents the interaction between two genes in the network. The edge weight corresponds to the PCC between the two genes. We identified the degree of gene centrality using the number of links from one node to the other in the network, which suggested its importance in biological function.

### RNA Isolation and Quantitative RT-PCR

Total RNA was extracted from cell lines and tissue samples using TRIzol Reagent (Takara, Tokyo, Japan) according to the manufacturer’s protocol and was reversed transcribed into cDNA using a Ribo^TM^ lncRNA qRT-PCR Starter Kit (Ribobio, Guangzhou, China). The reverse transcription (RT) protocol consisted of incubation at 42°C for 60 min followed by 70°C for 10 min. Quantitative real-time RT-PCR (qRT-PCR) analysis was performed in a total reaction volume of 20 μl, comprising 10 μl of SYBR Green Mix (2 X), 2 μl of cDNA template, 1 μl of lncRNA forward primer (5 μM), 1 μl of lncRNA reverse primer (5 μM), and 6 μl of RNA-free water. The qRT-PCR cycle settings included an initial denaturation step of 10 min at 95°C; 40 cycles of 5 s at 95°C and 30 s at 60°C, and a final extension step of 30 s at 72°C; reactions were performed with a Bio-Rad CFX96 instrument (ABI Corporation, CA, United States). The candidate lncRNAs were analyzed by qRT-PCR, and primers were purchased from Ribo Biotechnology (Guangzhou, China). We verified the expression of these lncRNAs using GAPDH as a housekeeping gene and by calculating the 2^–ΔΔCq^ values (Livak and Schmittgen, 2001). The primer sequences for qRT-PCR in our study were as follows: ENST00000456355 F: 5′-GGGCTGCCTTACCCTCATT-3′, R: 5′-GCCATACACTTTTAC ATCCTCCAT-3′; ENST00000594783 F: 5′-GGGGTTGAGACTG GGTGGAT-3′, R: 5′-TGCCTGGAATGTGTCTGTTTGT-3′; NONHSAT057283 F: 5′-TACTTATTTGCTTTCATGGGCC-3′, R: 5′-GGGAATGTGTGTTTAGTCTGCC-3′; ENST00000455354 F: 5′-TGATATCCGGACACATGGTGA-3′, R: 5′-CTGCAAAA ACGTGCTGAGAATT-3′; ENST00000422749 F: 5′-GCACGTT TTTGCAGAACCTG-3′, R: 5′-TCCCCTGTAGCGACACTGAG -3′; NONHSAT097797 F: 5′-CCGAAAGGATGCTGAACGC-3′, R: 5′-TCATGCTAAGCTGCCACTGC-3′.

### Overall Survival Analysis

The Kaplan–Meier Plotter database (Györffy et al., 2010)^4^ was used to confirm the prognostic power of the DE-mRNAs what were correlated with lnc-TRDMT1-5. To determine the potential prognostic value of lnc-TRDMT1-5 in BC patients, we downloaded the follow-up information of invasive BC patients from the TCGA dataset for overall survival (OS) analysis, and matched the expression profile of lnc-TRDMT1-5. Kaplan–Meier curves were analyzed by using SangerBox v1.0.9 (Mugu Biotech Company, Hangzhou, China); comparisons included the prognostic value of lnc-TRDMT1-5 (ENST00000456355) with ER and HER2 expression levels, and with patients who developed resistance to DOX during chemotherapy treatment. The hazard ratio (HR) and log rank *P* value are calculated and displayed.

### Analysis of the lncRNA–Target miRNA Network

PhyloCSF (Lin et al., 2011)^5^ was used to distinguish protein-coding and non-coding regions. lncLocator (Cao et al., 2018)^6^ was used to predict the subcellular location of lncRNAs based on sequence analysis, and to obtain the relative distributions to the cytoplasm, nucleus, ribosome, cytosol and exosome. The DIANA database (Paraskevopoulou et al., 2016)^7^ was used to predict target miRNAs of lncTRDMT1-5 based on the conserved sites matching the regions of lncRNA. The selected miRNAs were imported to TargetScan (Release 7.2),^8^ which can predict regulatory targets of miRNAs based on conserved sites. A cumulative weighted context ++ score ≤−0.2 was set as the threshold. After we screened for lncTRDMT1-5-correlated mRNAs, the lncTRDMT1-5-miRNA-mRNA network was constructed with Cytoscape.^9^

### Statistical Analysis

All data are shown as the means ± SDs and were analyzed using GraphPad Prism (version 7.0, CA, United States). Student’s *t* test was performed for comparisons between two groups, whereas ANOVA was performed for repeated measures. The χ^2^ test was used to analyze the correlation between lnc-TRDMT1-5 and clinical parameters. Differences with *P* < 0.05 were considered statistically significant. Fold change (FC) was used to analyze the statistical significance of the microarray results. FC ≥ 2 and *P* < 0.05 were considered the threshold values for designating DE-lncRNAs and DE-mRNAs.

## Results

### DE-lncRNAs and DE-mRNAs in DOX Resistant BC Cell Lines

**FIGURE 1:**
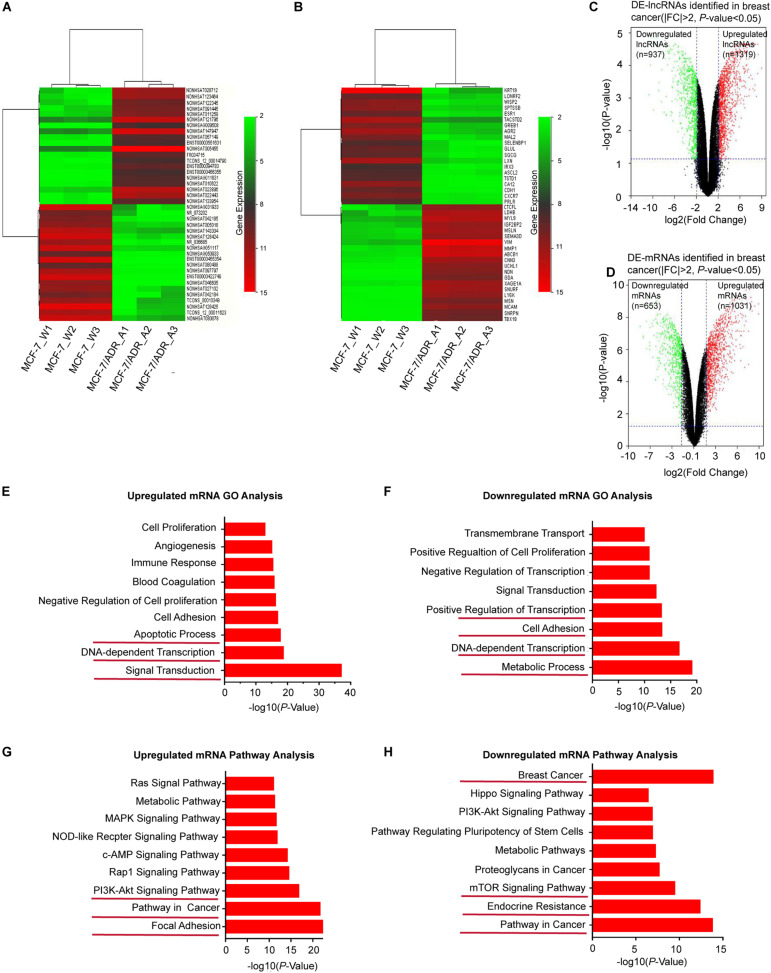
Overview of DE-lncRNAs and DE-mRNAs between MCF-7/ADR and MCF-7 cells in preparation of functional enrichment analysis. Heat map and hierarchical clustering of the top differentially expressed lncRNAs **(A)** and mRNAs **(B)** shared among the two group profiles (MCF-7/ADR and MCF-7 cells) in three biological replicates. The x-axis represents the sample names, and the y-axis represents different genes. Red indicates that a differentially expressed gene has a high expression value in the grouped sample, and green indicates that a differentially expressed gene has a low expression value. The top 40 DEGs are shown in the map according to the FCs. The magnitude of deviation from the median is represented by the color saturation. **(C)** Volcano plot showing all DE-lncRNAs in breast cancer. A total of 1319 significantly upregulated genes (red dots) and 937 downregulated genes (green dots) are highlighted for each time point (| FC| > 2, *P* < 0.05). **(D)** Volcano plot showing all DE-mRNAs in breast cancer. The red dots represent 1031 upregulated-mRNAs, while green dots represent 653 downregulated mRNAs (|FC| > 2, *P* < 0.05). **(E)** GO enrichment analysis of upregulated mRNAs. **(F)** GO enrichment analysis of downregulated mRNAs. The bar graphs represent the enrichment of these mRNAs. The value of (-logP) is the negative logarithm with base 10 of the P value. The threshold of significance was a *P* < 0.05, and the FDR was calculated to correct the *P*-value. **(G)** KEGG pathway enrichment analysis for upregulated mRNAs. **(H)** KEGG pathway enrichment analysis for downregulated mRNAs.

Based on the GSE81971 dataset, we analyzed the lncRNA and mRNA expression profiles in DOX-sensitive and DOX-resistant BC cells. Under the criteria of the | FC| ≥ 2, DE-lncRNAs and DE-mRNAs showed a variety of large quantities after reannotation with the lncRNA microarray V4 probes. Hierarchical clustering analysis showed that the expression patterns of the top 40 DE-lncRNAs (Figure 1A) and DE-mRNAs (Figure 1B) indicated a distinct signature. Among the top ranked lncRNAs and mRNAs in the clustering analysis, the lncRNAs NONHSAT005455, NONHSAT147947, and NONHSAT121796, and the mRNAs VIM, MMP1, and ABCB1 showed higher expression in MCF-7/ADR cells than in MCF-7 cells. In total, 2256 lncRNAs and 1684 mRNAs were found to be differentially expressed in the volcano plot, consisting of 1319 upregulated and 937 downregulated lncRNAs (Figure 1C), and 1031 upregulated and 653 downregulated mRNAs (Figure 1D). All of these DE-RNAs might participate in the functional processes of DOX-resistant BC.

### Functional Enrichment Analysis of DE-mRNAs

To investigate the key biological functions associated with the DE-RNAs, we performed functional enrichment analysis on the upregulated and downregulated genes. DE-lncRNAs can be associated with biological pathways by applying their association with mRNAs. Therefore, the expression profile of all DE-mRNAs was integrated to identify associated pathways. GO analysis revealed that upregulated mRNAs were significantly enriched in transcriptional processes, such as signal transduction, DNA-dependent transcription, and apoptotic signaling (Figure 1E), while downregulated mRNAs were involved in metabolic processes, cell adhesion, and transcription, including DNA-dependent transcription and positive regulation of transcription (Figure 1F). Furthermore, KEGG pathway analysis indicated that upregulated mRNAs were enriched in cancer-related pathways, such as focal adhesion and the PI3K/AKT signaling pathway (Figure 1G), while downregulated mRNAs were enriched in resistance-related pathways, such as endocrine resistance and the mTOR signaling pathway (Figure 1H). Based on these identified biological processes and pathways, we aimed to screen for DE-mRNAs that overlapped in the GO and KEGG pathway enrichment analysis; 495 mRNAs with significant differences (*P* < 0.05) were selected for further analysis. Taken together, these findings indicated that these DE-mRNAs may play functional roles in BC tumorigenesis and chemoresistance. Thus, we hypothesized that the lncRNAs associated with these mRNAs might also be involved in the same functional processes.

### Construction of an lncRNA-mRNA Coexpression Network

**FIGURE 2:**
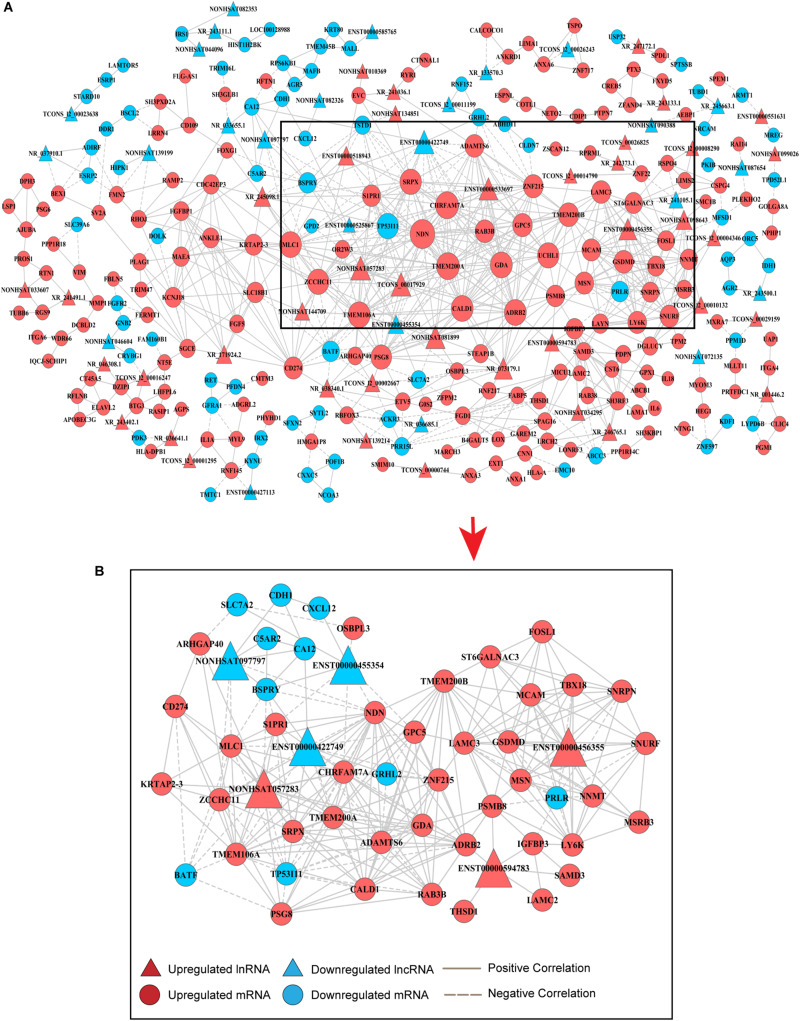
Coexpression network of DE-lncRNAs and DE-mRNAs for predicting the functional roles of candidate lncRNAs. **(A)** Overview of the coexpression network comprising lncRNA associations with mRNAs. The key genes in the center of the network are in the black triangle area. The red colored genes represent upregulated lncRNAs and mRNAs, while the blue color indicates downregulated genes. Positive correlations are represented by a solid line, and negative correlations are represented by a dotted line. Data are expressed using the comparative cycle threshold method. **(B)** The subnetwork in the black area above was selected to show the relationship between candidate hub lncRNAs and mRNAs.

An lncRNA-mRNA coexpression network was used to identify the interactions between mRNAs and lncRNAs, elucidate the roles of lncRNAs and screen for hub lncRNAs. Here, we constructed an lncRNA-mRNA coexpression network based on the correlation analysis between lncRNAs and the 495 overlapping mRNAs. Based on the normalized signal intensities, which represent the expression level of specific mRNAs and lncRNAs, a PCC equal to or greater than 0.99 was used to identify interactions between lncRNAs and mRNAs. A total of 855 pairs of coexpressed lncRNAs and mRNAs were involved and used to depict their relationships, consisting of 245 mRNAs and 67 lncRNAs (Supplementary Table S1). These RNA molecules were interregulated, and most of these lncRNA–mRNA coexpression relationships showed a positive correlation, suggesting that these lncRNAs were involved in crucial functions in drug-resistant BC cells. Additionally, one lncRNA was correlated with several mRNAs and one mRNA was coexpressed with more than one lncRNA, further supporting the notion of interregulatory relationships. To identify candidate lncRNAs among these DE-lncRNAs, we constructed lncRNA–mRNA coexpression patterns based on the correlation analysis (Figure 2A).

Degree centrality is defined as the number of links one node has to another, and it is the simplest and most important measure of gene or lncRNA centrality within a network and determines the relative importance of the nodes (Barabási and Oltvai, 2004). The candidate DE-lncRNAs were selected based on higher degrees (degree ≥ 6), which lay in the central location of the coexpression network. From the center of the network, we chose three upregulated DE-lncRNAs (ENST00000456355, ENST00000594783 and NONHSAT057283) and three downregulated DE-lncRNAs (ENST00000455354, ENST00000422749, and NONHSAT097797) and reorganized a clear and direct lncRNA-mRNA coexpression network for further analysis (Figure 2B). Among these lncRNAs, ENST00000422749, NONHSAT057283, and ENST00000456355 shared significant correlations with many more mRNAs. In Table 1, we provide a detailed description of the FC of these six candidate lncRNAs and their correlated mRNAs, which might enable their participation in diverse signaling pathways. These mRNAs were likely to be the potential targets, or sponsors, of the six candidate lncRNAs. The known lncRNA ENST00000456355 (LNCipedia: lnc-TRDMT1-5, AL133415.1), was most correlated with melanoma cell adhesion molecule (MCAM), T-box transcription factor 18 (TBX18), small nuclear ribonucleoprotein polypeptide N (SNRPN), moesin gene (MSN), nicotinamide N-methyltransferase (NNMT), FOS-related antigen 1 (FOSL1), and prolactin receptor (PRLR). The known lncRNA ENST00000594783 (ZNF667-AS1) was correlated with thrombospondin type-1 domain-containing protein 1 (THSD1), insulin-like growth factor binding protein-3 (IGFBP3), laminin subunit gamma-2 (LAMC2), sterile alpha motif domain containing 3 (SAMD3) and arrestin beta 2 (ARB2). NONHSAT057283 (lnc-MPPE1-13) was correlated with zinc finger CCHC domain containing 11 (ZCCHC11), TP53, modulator of VRAC current 1 (MLC1), CD274 and sushi repeat containing protein X-linked (SRPX). ENST00000455354 (DSCAM-AS1:5) was correlated with sphingosine-phosphate receptor 1 (S1PR1), solute carrier family 7 member 2 (SLC7A2) and necdin (NDN). ENST00000422749 (DSCAM-AS1:2) was correlated with transmembrane protein 200A (TMEM200A), NDN, zinc finger protein 215 (ZNF215), and ADRB2. NONHSAT097797 (lnc-CFI-3) was correlated with ZCCHC11, NDN, and cadherin 1 (CDH1). Notably, the DE-mRNAs MCAM, SNRPN, FOSL1, and IGFBP3 were strongly correlated with each other, providing us with a highly integrated lncRNA–mRNA coexpression network.

**TABLE 1:** Correlated analysis between six candidate DE-lncRNAs and mRNAs.

DE-lncRNAs	Fold change	*P*-value	Level	Degree	Correlated mRNAs	*R* value
ENST 00000422749	−281.293	3.70E-05	DOWN	18	ZNF215	–0.99996
(DSCAM-AS1:2)					ZCCHC11	–0.99998
					TP53I11	0.999956
					TMEM200A	–0.999985
					TMEM106A	–0.999953
					SRPX	–0.999975
					RAB3B	–0.999951
					NDN	–0.99998
					MLCl	–0.999973
					GRHL2	–0.99996
					GPC5	–0.999953
					GDA	–0.999962
					CHRFAM7A	–0.999962
					CALDl	–0.99997
					CA12	–0.999957
					BSPRY	–0.999956
					ADRB2	–0.999954
					ADAMTS6	–0.999972
NONHSAT057283	13.59	0.00076	UP	18	ZCCHC11	0.999978
(lnc-MPPEl-13)					TP53I11	–0.999961
					TMEM200A	0.999963
					TMEM106A	0.999986
					SRPX	0.999956
					SlPRl	0.99998
					PSG8	0.999971
					NDN	0.999989
					MLCl	0.999981
					KRTAP2-3	0.999948
					GDA	0.999959
					CHRFAM7A	0.999962
					CD274	0.999957
					CALDl	0.999954
					BSPRY	–0.999964
					BATF	–0.999951
					ARHGAP40	0.999979
					ADRB2	0.999951
ENST00000456355	60.836	0.00022	UP	14	TMEM200B	0.999954
(Lnc-TRDMTl-5)					TBX18	0.999978
					ST6GALNAC3	0.999984
					SNURF	0.999966
					SNRPN	0.999982
					PRLR	–0.999955
					NNMT	0.999974
					MSRB3	0.99996
					MSN	0.99997
					MCAM	0.999991
					LY6K	0.999974
					LAMC3	0.999994
					GSDMD	0.999986
					FOSL1	0.999984
ENST00000455354	−1394.049	3.4E-05	DOWN	8	SLC7A2	0.999956
(DSCAM-AS1:5)					S1PR1	–0.999981
					OSBPL3	–0.999963
					NDN	–0.999972
					GPC5	–0.999974
					CXCL12	0.999957
					CHRFAM7A	–0.999951
					BSPRY	0.999960
ENST00000594783	109.815	0.00011	UP	6	THSD1	0.999953
(ZNF667-AS1)					SAMD3	0.999954
					PSMB8	0.999981
					LAMC2	0.999971
					IGFBP3	0.999962
					ADRB2	0.999951
NONHSAT097797	−537.10388	4.3E-05	DOWN	7	ZCCHC11	–0.999963
(lnc-CFI-3)					NDN	–0.999949
					MLC1	–0.999965
					CDH1	0.999954
					CA12	0.999994
					C5AR2	0.999978
					BSPRY	0.999986

*DE-lncRNA, difterentially expressed IncRNAs; Degree, node degree value; R value, coefficient of correlation. R value > 0 equals to positive relationship; R value < 0 equals to negative relationship.*

### lncRNA-mRNA-Pathway Network

**FIGURE 3:**
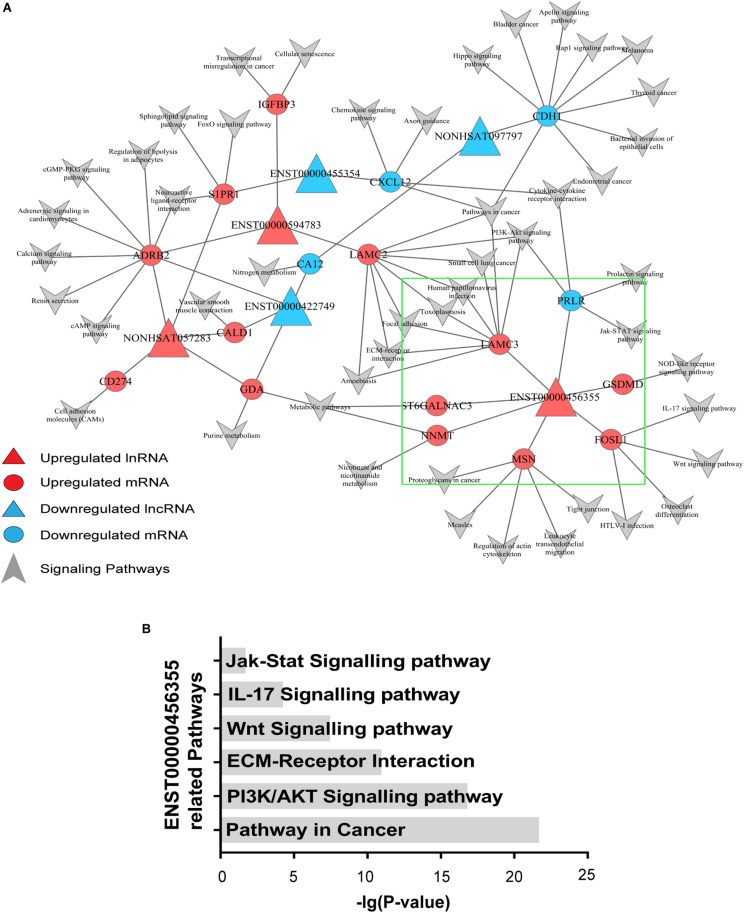
Signaling pathway analysis of the lncRNAs. **(A)** An lncRNA–mRNA-pathway network was constructed with the six selected lncRNAs. Triangle nodes represent dysregulated lncRNAs, and circle nodes represent dysregulated mRNAs. The red nodes of lncRNAs and mRNAs indicate upregulated expression, while the blue nodes indicate downregulated expression. The arrows refer to the signaling pathways in which lncRNAs and mRNAs might participate. The lncRNAs in the red triangle area with larger nodes located in the center of the network indicate the key lncRNAs in the regulated network. **(B)** Pathways associated with lnc-TRDMT1-5 were found to be significant based on the *P*-value of lnc-TRDMT1-5.

To explore the signaling pathways in which the six candidate lncRNAs are involved, we constructed an lncRNA–mRNA-pathway network, which showed that the expression of one mRNA may correlate with several lncRNAs, while the expression of one lncRNA may correlate with different mRNAs. Notably, different mRNAs might participate in the same signaling pathways. For instance, PRLR expression was negatively correlated with lnc-TRDMT1-5, and it probably functioned through the PI3K/AKT and Jak/Stat signaling pathways, while LAMC2 expression positively correlated with that of ENST00000594783 but could also function through the PI3K/AKT signaling pathway. Additionally, S1PR1 expression, which was correlated with that of ENST00000455354 and NONHSAT057283, probably functioned through the FoxO signaling pathway. ADRB2 expression, which was positively correlated with that of ENST00000594783 and NONHSAT057283, functions in the c-AMP signaling pathway (Figure 3).

In the area indicated by a green triangle, a coexpression network revealed that six mRNAs shared a positive correlation with lnc-TRDMT1-5 whereas only one mRNA shared a negative relationship with lnc-TRDMT1-5 (Figure 3A). In detail, FOSL1 was closely related to the Wnt and IL-17 signaling pathways, and GSDMD functioned via the NOD-like receptor signaling pathway. Mechanistically, six pathways with FDRs < 0.05 were significantly enriched with mRNAs that were coexpressed with lnc-TRDMT1-5; those pathways included the PI3K/AKT signaling pathway (*P* = 2.02 × 10^–17^), ECM-receptor interaction (*P* = 1.54 × 10^–11^) and Jak/STAT signaling pathway (*P* = 0.02) (Figure 3B).

**FIGURE 4:**
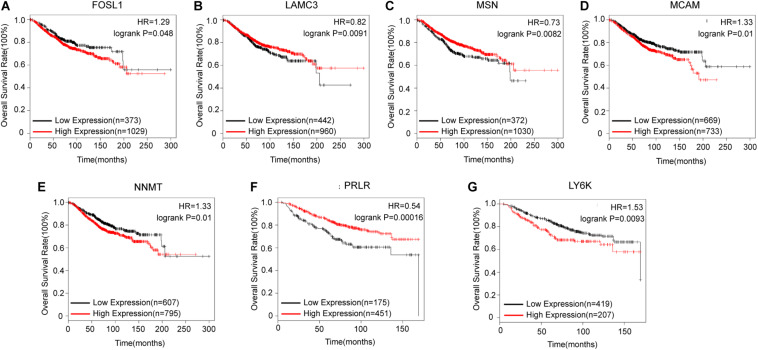
Kaplan–Meier survival curves for mRNAs correlated with lnc-TRDMT1-5 (n = 1402). **(A)** Survival curves for FOSL1. **(B)** Survival curves for LAMC3. **(C)** Survival curves for MSN. **(D)** Survival curves for MCAM. **(E)** Survival curves for NNMT. **(F)** Survival curves for PRLR. **(G)** Survival curves for LY6K. Horizontal axis, survival time (days); vertical axis, survival rate. HR: Hazard ratio.

After identifying coexpressed lncRNA-mRNAs, KM plotter analysis was performed to examine whether these mRNAs were associated with the outcomes of BC patients. Furthermore, we identified that high expression of FOSL1, MCAM, NNMT, and LY6K, and low expression of LAMC3, MSN, and PRLR were correlated with poor prognosis of BC based on patients from the TCGA database (Figure 4). Thus, these results indicated that lnc-TRDMT1-5 might participate in these key kinase signaling pathways and affect BC prognosis.

### Analysis of lnc-TRDMT1-5 Using RT-PCR Analysis in BC Tissues

**FIGURE 5:**
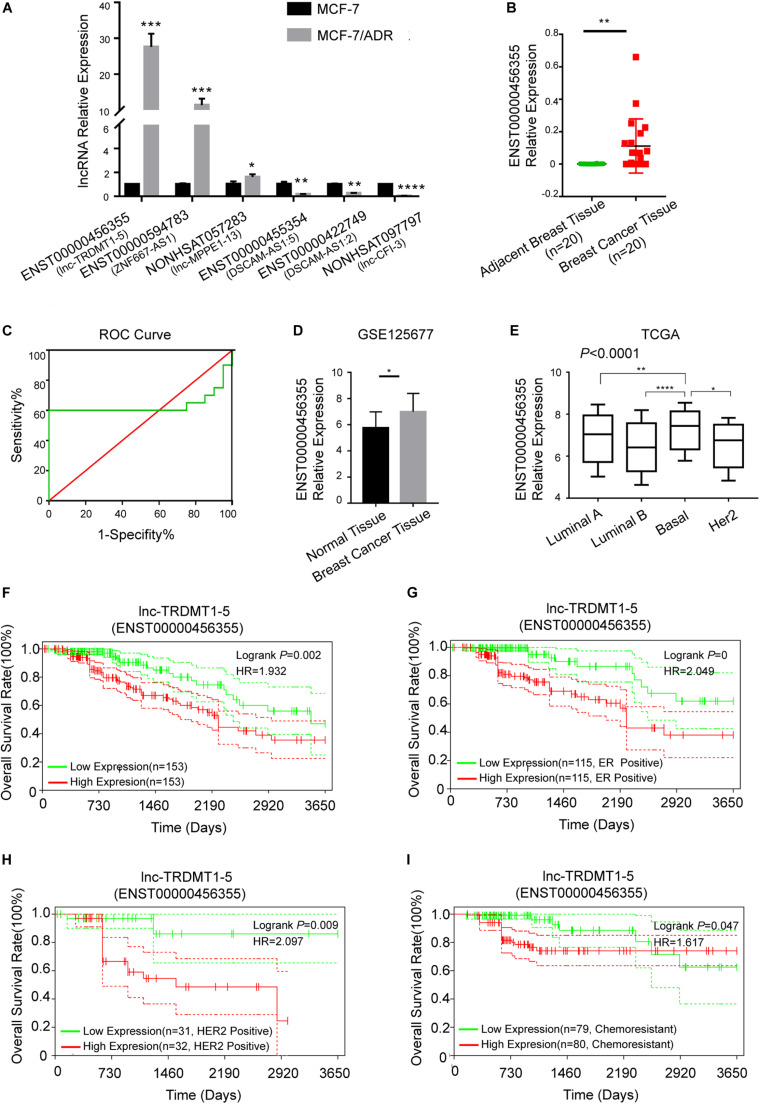
Lnc-TRDMT1-5 was upregulated in BC and could serve as a vital biomarker. **(A)** Quantitative PCR analysis of the six hub lncRNAs was performed to identify significant differences between MCF-7/ADR and MCF-7 cells. **(B)** The expression level of lnc-TRDMT1-5 was increased in BC tissues (*n* = 20) compared to adjacent tissues (*n* = 20). **(C)** ROC curve analysis of lnc-TRDMT1-5 expression in the 20 pairs of BC samples. The abscissa indicates 100% specificity, and the ordinate indicates sensitivity. **(D)** The expression level of lnc-TRDMT1-5 in BC tissues was compared to that in normal tissues from the GSE125677. **(E)** Differential expression levels of lnc-TRDMT1-5 in four molecular subtypes of BC from the TCGA database (588 invasive BC tissue samples and 55 normal samples). **(F)** Kaplan-Meier survival curves for lnc-TRDMT1-5 in BC patients whose follow-up data and the lnc-TRDMT1-5 expression data were available (*n* = 306). **(G)** Survival curves for ER + BC patients (*n* = 230). **(H)** Survival curves for HER2 + patients (*n* = 63). **(I)** Survival curves for chemoresistant patients (*n* = 159). Horizontal axis: survival time (days); vertical axis: survival rate. HR: Hazard ratio. Red and blue dashed lines in the figure indicate confidence intervals. *P < 0.05, **P < 0.01, ****P < 0.00001.

To further verify the functional role of the six candidate lncRNAs related to cancer progression, we validated the expression level of these lncRNAs in MCF-7 and MCF-7/ADR cells that were cultured in our laboratory. We confirmed that the lnc-TRDMT1-5 expression level was dramatically increased in MCF-7/ADR cells compared with MCF-7 cells. The results were consistent with those of the GSE81971 dataset (Figure 5A). The results of the other five lncRNAs were also consistent with those of the GSE81971 dataset. Next, qRT-PCR analysis was performed on 20 pairs of BC tissues and their corresponding adjacent tissues, and the results showed that the expression level of lnc-TRDMT1-5 was upregulated in BC tissues compared with adjacent tissues. Receiver operating characteristic (ROC) curve analysis showed that the area under the curve was greater than 0.5 (Figure 5C). In the GSE125677 dataset, we also found that the expression level of lnc-TRDMT1-5 was higher in BC tissues than in normal tissues (Figure 5D). Otherwise, the expression level of lnc-TRDMT1-5 was significantly variable in the different subtypes of invasive BC from the TCGA dataset (Figure 5E). The lnc-TRDMT1-5 expression level was relatively higher in the basal type than in the luminal A or B type or HER2-positive type. However, the lnc-TRDMT1-5 level among the luminal A, luminal B, and HER2-potive subtypes showed no significant difference (*P* > 0.05). Furthermore, the correlation analysis between lnc-TRDMT1-5 expression and the clinicopathological characteristics illustrated that lnc-TRDMT1-5 levels were significantly increased in ER-positive and HER2-positive BC patients (Table 2) and that lnc-TRDMT1-5 expression may be potentially related to TNM stage and the level of Ki67 positivity (*P* = 0.051). However, lnc-TRDMT1-5 expression showed no significant correlations with either age or lymphatic metastasis. These results indicate that lnc-TRDMT1-5 is probably involved in BC development and chemoresistance, and its expression level is related to ER and HER2 levels. These data also confirmed the results of the microarray analysis, indicating that lnc-TRDMT1-5 might serve as an oncogenic biomarker in BC progression.

**TABLE 2:** Association between ENST00000456355 and clinicopathological characteristics of patients with breast cancer (*n* = 20).

	*n* = 20	ENST00000456355 expression		χ^2^	*P*-value
		High	Low		
**Age**					
>45	15	9	6	0.606	0.436
≤45	5	2	3		
**Lymphnode metastasis**					
Yes	8	6	2	3.333	0.068
No	12	3	9		
**ER**					
Positive	12	9	3	4.848	0.027*
Negative	8	2	6		
**PR**					
Positive	10	7	3	1.818	0.178
Negative	10	4	6		
**HER2**					
Positive	14	12	2	5.488	0.019*
Negative	6	2	4		
**TNM stage**					
T1/T2	14	5	9	3.810	0.051
T3/T4	6	5	1		
**Ki-67**					
≤10%	6	2	4	3.778	0.051
>10%	14	11	3		

*p < 0.05.

### The Prognostic Value of lnc-TRDMT1-5 in BC Patients

Biological factors that influence BC survival include tumor stage, tumor grade, ER and progesterone receptor (PR) status, and HER2 status (Miller et al., 2019). Based on the clinicopathological characteristics analysis, we evaluated the OS analysis in BC patients with different ER status, HER2 status and different outcomes of chemotherapy. First, the follow-up data of patients with all pathological types of BC were downloaded from the TCGA dataset, and then we matched the lnc-TRDMT1-5 expression profile downloaded from 587 invasive BC patients from the TCGA database to detect its expression levels (Figure 5E; total number of BC patients was 589, two duplicates were removed) with those samples containing the follow-up survival data. A total of 572 BC samples got both lnc-TRDMT1-5 expression levels and the follow-up survival data, and 306 BC samples were identified by SangerBox software for OS analysis. The results showed that high expression level of lnc-TRDMT1-5 predicted a poor overall survival rate in BC patients (Figure 5F). Based on our above hypothesis, these results showed that the high lnc-TRDMT1-5 expression was associated with poor prognosis regardless of ER+ status (Figure 5G), HER2+ status (Figure 5H), and chemoresistance status (Figure 5I). However, aberrant expression of lnc-TRDMT1-5 had no significant effect on the overall survival rate of ER-negative patients (log-rank *P* = 0.61, Supplementary Figure S1A), and it was necessary to increase the number of patients with available clinical data before further survival analyses can be conducted. To evaluate the expression of lnc-TRDTM1-5 and the overall survival rate for ER-positive and ER-negative patients, we compared the overall survival rate between ER-positive and ER-negative patients in the lnc-TRDMT1-5 expression profile from the TCGA database, the results showed that ER-positive patients had a significantly higher survival rates as compared to ER-negative patients (Supplementary Figure S1B). In addition, the expression level of lnc-TRDTM1-5 in ER-negative tissues was higher than that in ER-positive patients (Supplementary Figure S1C). We also determined the expression level of lnc-TRDMT1-5 in ER-positive and ER-negative patients in our collected patient samples. Unfortunately, there was no significant difference in the lnc-TRDTM1-5 expression level between these two groups (Supplementary Figure S1D). However, these results indicated a correlation between lnc-TRDMT1-5 and ER levels. We speculated that the high expression of lnc-TRDMT1-5 in ER-negative patients may be one of the potential factors for poor survival of BC patients, which suggested that lnc-TRDMT1-5 could be a useful prognostic biomarker in BC.

### The Predictive ceRNA Activity of lnc-TRDMT1-5

**FIGURE 6:**
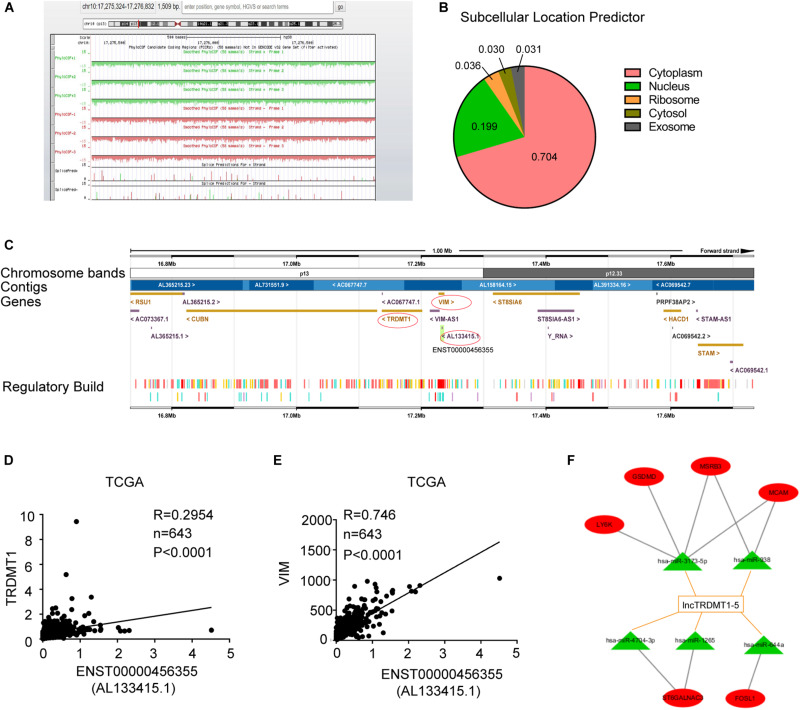
Subcellular location and functional ceRNA network prediction for lnc-TRDMT1-5. **(A)** Schematic annotation of the lnc-TRDMT1-5 genomic locus on chromosome 10q17,275,324-17,276,832 in humans. Coding potency was analyzed using PhyloCSF. Scores above zero suggest coding potential, whereas scores below zero represent non-coding potential. **(B)** The pie chart shows the subcellular location scores of lnc-TRDMT1-5 using a subcellular location predictor (lncLocator) (http://www.csbio.sjtu.edu.cn/bioinf/lncLocator/) according to the sequence analysis. **(C)** The region of ENST0000456355 (AL133415.1) was identified in detail by the Ensembl database (http://asia.ensembl.org), and it shows that the protein coding gene VIM shared part of the location site with AL133415.1. Another coding gene, TRDMT1, was upstream of AL133415.1. **(D)** A correlation analysis between lnc-TRDMT1-5 and TRDMT1 in the TCGA database was performed on 590 invasive BC and 53 normal breast tissues. **(E)** The correlation analysis between lnc-TRDMT1-5 and VIM was performed on 590 invasive BC and 53 normal breast tissues. **(F)** A correlation network comprising lnc-TRDMT1-5-miRNA-mRNA. Correlation between lncTRDMT1-5 and miRNAs were screened for the criteria of correlation scores >0.9, and binding scores >0.04. Correlations between selected miRNAs and mRNAs were calculated and identified by using the TargetScan database. A total of five miRNAs and six mRNAs were selected with connecting edges using Cytoscape 3.6.

To further analyze the transcriptional regulation of lnc-TRDMT1-5, we performed bioinformatics analysis to assess its coding potential and subcellular location. After entering the location information of lnc-TRDMT1-5, we observed that the PhyloCSF value was negative (the green and red areas were all below the black baseline), revealing that lnc-TRDMT1-5 was definitely a non-coding gene (Figure 6A). lncLocator was used to obtain the distribution ratio, and lnc-TRDMT1-5 was mostly localized to the cytoplasm (70.4%) (Figure 6B), suggesting that lnc-TRDMT1-5 mainly functioned in the cytoplasm, likely via the competing endogenous RNA (ceRNA) regulatory mechanism. Moreover, the lnc-TRDMT1-5 sequence was analyzed in the Ensembl database^10^ to identify neighboring genes that lay up- and downstream (Figure 6C). Surprisingly, we found that the TRDMT1 gene was located upstream of gene VIM, while VIM shared partial sequences with lnc-TRDMT1-5. These findings suggested that TRDMT1 and VIM may act as cis-regulated genes, promoting lnc-TRDMT1-5 expression levels. To investigate the correlation between these genes, we examined their expression levels in invasive BC samples from the TCGA database, and Spearman correlation analysis between these genes suggested that lnc-TRDMT1-5 was positively correlated with TRDMT1 (Figure 6D) and VIM (Figure 6E), while the correlation coefficient between lnc-TRDMT1-5 and VIM (*R* = 0.746) was higher than that between lnc-TRDMT1-5 and TRDMT1 (*R* = 0.295). To further investigate the functional ceRNA network, an lncRNA-miRNA network was constructed using the DIANA-LncBase V.2 database. The results showed that miR-3173-5p, miR-938, miR-4704-3P, miR-1265, and miR-644a possibly interacted with lnc-TRDMT1-5. We also predicted the biological targets of these miRNAs using TargetScan Release 7.2. After integrating the data from these databases, we constructed a correlation network and found that there was a potential correlation between LY6K, GSDMD, MSRB3, MCAM, ST6GALNAC3, and FOSL1 with different miRNAs and lnc-TRDMT1-5. Taken together, these findings suggest that cytoplasmic lncTRDMT1-5 might participate in a ceRNA regulatory network.

## Discussion

Genome-wide studies have revealed that there are more non-coding RNAs than protein coding RNAs (Sun et al., 2018). LncRNAs have been found to be involved in various types of cancer, where they act as either oncogenes or tumor suppressors (Bhan et al., 2017). However, a large number of lncRNAs involved in BC remain unexplored.

In this study, we identified DE-lncRNAs and -mRNAs based on the GSE81971 dataset, and then we constructed an lncRNA–mRNA-pathway network linked to BC cell lines with DOX resistance. In accordance with the study based on the GSE81971dataset (He et al., 2016), large numbers of DE-lncRNAs and DE-mRNAs were screened. Unlike their approach in choosing the top DE-lncRNAs, we looked for functional mRNAs that overlapped in the GO and pathway enrichment analyses to find the correlated lncRNAs, despite the marked alteration in a large number of DE-lncRNAs and DE-mRNAs. The lncRNA-mRNA coexpression network we constructed clearly revealed that some crucial lncRNAs and mRNAs act as important regulators in a wide range of biological functions, and it also provided a new view of potential lncRNAs involved in BC chemoresistance. Specifically, a total of 312 lncRNAs and 856 mRNAs were differentially expressed in resistant cells compared with sensitive cells, and shared correlations were used to generate a coexpression network. Furthermore, we found that these DE-mRNAs were involved in transcript regulation, and the phosphoinositide 3-kinase (PI3K)/Akt/mTOR signaling pathway, both of which play important roles in BC tumorigenesis (Guerrero-Zotano et al., 2016), progression, and chemoresistance (Okada et al., 2015). Targeting these pathways has been shown to augment the benefits of endocrine therapy in ER-positive BC (Ciruelos Gil, 2014), and improve clinical outcomes (Yang et al., 2016). We identified six hub lncRNAs, the expression of which correlated with different DE-mRNAs. Recent studies have shown that some of these DE-mRNAs, such as NDN (Lee et al., 2015), S1PR1 (Nagahashi et al., 2018) and ADRB2 (Gargiulo et al., 2014), are involved in cancer progression, and ZCCHC11, MLC1, CALD1, mutated TP53, and CD274 are uniquely regulated by the let-7 regulatory pathway (Qian et al., 2016), the NF-κB signaling pathway (De Marchi et al., 2016), and the PI3K/AKT pathway (Alsuliman et al., 2015). TMEM106A, acts as a tumor suppressor and is regulated by promoter hypermethylation in gastric cancer cells (Xu et al., 2014), and FOSL1 has been linked to primary breast tumor metastasis (Callegari et al., 2016). Moreover, the nuclear localization of IGFBP3, which is highly expressed in ER-negative BC cells, was enhanced by treatment with DOX, suggesting that targeting IGFBP3 is a therapeutic approach for sensitizing BC cells to chemotherapy (Lin et al., 2014). Therefore, all these mRNAs might have prominent roles in BC tumorigenesis and acquired chemoresistance (Williams and Cook, 2015), the dysregulation of which predicts poor overall survival. Taken together, the data related to the coexpression network shown here will potentially lead to a better understanding of the functional mechanisms of dysregulated lncRNAs.

We identified key lncRNAs with a high degree of confidence, and found that lnc-TRDMT1-5 and ZNF667-AS1 were the top two upregulated lncRNAs in the subnetwork. We further identified that PRLR was negatively associated with lnc-TRDMT1-5, and that LAMC2 was positively associated with ZNF667-AS1. Enrichment analysis revealed that these two mRNAs participated in the PI3K/AKT signaling pathway, indicating that lncTRDMT1-5 and ZNF667-AS1 may participate in molecular pathogenesis via transcriptional regulation, where they could regulate AKT phosphorylation and cAMP-response element binding protein (CREB) activation (Phuong et al., 2014). Studies have shown that ZNF667-AS1 serves as a potential target for antitumor therapy and is potentially correlated with the prognosis of esophageal squamous cell carcinoma (Dong et al., 2019) and gastric cancer (Peng et al., 2020). Thus, we chose another potential lncRNA, lncTRDMT1-5, whose function and biological mechanism have not been elucidated and validated by experimental studies. Our lncRNA–mRNA coexpression network indicates that lnc-TRDMT1-5 and its coexpressed mRNAs act as important contributors to chemoreistance in BC.

A comprehensive functional analysis of lncRNAs was performed via specific experiments to confirm the prediction results obtained by bioinformatics analysis. The expression level of lnc-TRDMT1-5, a 706 bp non-coding transcript, was shown to be higher in BC tissues compared with adjacent normal tissues and in chemoresistant cancer cells compared with sensitive cells, indicating that it might be involved in cancer development and drug resistance. Based on the tissue samples we collected, clinicalpathologic analysis showed the close relationships between lnc-TRDMT1-5 and age, TNM stage, hormone receptor levels, etc. Additionally, our findings indicated that lnc-TRDMT1-5 expression was strongly correlated with ER and HER2 expression levels. Due to the small sample size of tissues we collected, we also analyzed lnc-TRDMT1-5 expression in the TCGA database. Although the lnc-TRDMT1-5 expression was decreased in BC tissues compared with normal breast tissues (data not shown), but ER-positive samples exhibited higher lnc-TRDMT1-5 expressions than ER-negative samples. However, the expression level of lnc-TRDMT1-5 in ER-positive and ER-negative samples in the TCGA database were quite different from our results, the main reason was that our sample numbers were much lower than the TCGA database, and more tissue samples were required to verify these results. It is still worth noting that the lnc-TRDMT1-5 expression was profoundly varied among the basal, luminal A/B and HER2-positive subtypes of BC in the TCGA database, indicating that the level of lnc-TRDMT1-5 expression is potentially related to expression levels of ER and HER2, which were consistent with our analyses of clinical characteristic. In addition, we observed that upregulation of lnc-TRDMT1-5 was correlated with poor prognosis in patients with ER-positive, HER2-positive, and chemoresistant BC. Compared with ER-negative patients, ER-positive patient samples exhibited better prognosis and lower expression levels of lnc-TRDMT1-5. These findings indicated that lnc-TRDMT1-5 could be considered a pathological and prognostic factor. The survival analysis software will automatically remove the expression value which equals to 0, and the survival time which is less than 30 days, more tissue samples were required to verify the survival rate in ER-negative patients. We will conduct further functional experiments with more BC samples in the future.

The molecular function of lncRNAs depends on their subcellular localization, and cytoplasmic lncRNAs have been proven to play diverse roles in regulating translation and signaling (Carlevaro-Fita and Johnson, 2019), as well as acting as ceRNAs, the mechanisms of which often involve regulatory relationships with lncRNAs, miRNAs, and mRNAs; furthermore, they have critical roles in establishing the hallmarks of BC development (Abdollahzadeh et al., 2019). Cis-acting lncRNAs have been demonstrated to activate, repress or modulate the expression of target genes (Gil and Ulitsky, 2019). In the genomic map, TRDMT1 and VIM, which are located upstream of lnc-TRDMT1-5, might act as cis-regulated genes, promoting lnc-TRDMT1-5 expression levels. Our prediction suggests that lncTRDMT1-5 functions through competitive binding to response elements of miRNAs (e.g., miR-3173-5P, miR-938, miR-4704-3P, miR-1265, and miR-644a). Furthermore, an lncRNA–miRNA–mRNA network illustrated direct interactions to help us elucidate the complex mechanism by which these genes are associated with drug resistance in BC, but these results still need to be experimentally validated.

In summary, we identified crucial DE-lncRNAs and DE-mRNAs and selected candidate lncRNAs through a coexpression network, but the detailed mechanism underlying chemoresistance remains unclear. We constructed an lncRNA–mRNA network linked to DOX resistance, but other regulation mechanisms might exist. Our results provide basis for subsequent verification on the highlighted lncRNAs, but we are not limited to this result. To elucidate the functional analysis of lnc-TRDMT1-5, overexpression and knockdown experiments will be performed to identify the relationship between lncRNAs and mRNAs. In addition, more tissue samples are required for further research. Therefore, our study revealed that lnc-TRDMT1-5 is involved in chemoresistance in BC, and might play a significant role in tumorigenesis via ceRNA regulation, which makes the molecule a potential diagnostic and prognostic biomarker for BC.

## Data Availability Statement

The gene expression profile of GSE81971 was downloaded from Gene Expression Omnibus datasets (GEO, http://www.ncbi.nlm.nih.gov/geo/).

## Ethics Statement

This study was carried out in accordance with the recommendations of the Ethics Committee of Fourth Affiliated Hospital of Jiangsu University (No. 201809). The protocol was approved by the Ethics Committee of Fourth Affiliated Hospital of Jiangsu University. All subjects gave written informed consent in accordance with the Declaration of Helsinki.

## Author Contributions

WX and HC contributed the conception and design of the study. HY collected tissue samples. SX organized the database. QC and XZ performed the statistical analysis. QC wrote the first draft of the manuscript. All authors contributed to manuscript revision, read and approved the submitted version.

## Conflict of Interest

The authors declare that the research was conducted in the absence of any commercial or financial relationships that could be construed as a potential conflict of interest.

## Footnotes

1 http://www.ncbi.nlm.nih.gov/geo/

2 www.lncipedia.org

3 http://david.abcc.ncifcrf.gov/tools.jsp

4 http://kmplot.com/analysis/index.php?p=service&cancer=breast

5 http://genome.ucsc.edu

6 http://www.csbio.sjtu.edu.cn/bioinf/lncLocator/

7 http://carolina.imis.athena-innovation.gr/diana_tools/web/index.php

8 http://www.targetscan.org/

9 http://www.cytoscape.org/

10 http://asia.ensembl.org

## Abbreviations

BC: breast cancer
DAVID, Database for Annotation: Visualization and Integrated Discovery
DEGs: differentially expressed genes
DE-lncRNAs: differentially expressed lncRNAs
DE-mRNAs: differentially expressed mRNAs
DOX or ADR: doxorubicin
ER: estrogen receptor
FC: fold change
FDR: false positive rate
GEO: Gene Expression Omnibus
HR: hazard ratio
HER2: human epidermal growth factor receptor 2
lncRNAs: long non-coding RNAs
NGS: next-generation sequencing
OS: overall survival
PCC: Pearson’s correlation coefficient
TNBC: triple negative breast cancer.
