# Supplementary material for: Integrative Analysis of the Doxorubicin-Associated LncRNA–mRNA Network Identifies Chemoresistance-Associated lnc-TRDMT1-5 as a Biomarker of Breast Cancer Progression
Source: Front Genet. 2020 May 29;11:566. doi: 10.3389/fgene.2020.00566 (PMC7272716; doi:10.3389/fgene.2020.00566)
Supplement: Supplementary file 1 [file Table_1.DOCX]

| **Supplementary Table S1.** Correlation analysis between mRNAs-mRNAs and lncRNAs-mRNAs. | | | | | |  |
| --- | --- | --- | --- | --- | --- | --- |
| Gene_1 | Biotype_1 | Gene_2 | Biotype_2 | prediction | R value | Type |
| GRHL2 | mRNA | ENST00000422749 | ncRNA | mRNA-ncRNA | 0.999960 | 1 |
| CA12 | mRNA | ENST00000422749 | ncRNA | mRNA-ncRNA | 0.999957 | 1 |
| TP53I11 | mRNA | ENST00000422749 | ncRNA | mRNA-ncRNA | 0.999956 | 1 |
| BSPRY | mRNA | ENST00000422749 | ncRNA | mRNA-ncRNA | 0.999956 | 1 |
| RAB3B | mRNA | ENST00000422749 | ncRNA | mRNA-ncRNA | -0.999951 | -1 |
| TMEM106A | mRNA | ENST00000422749 | ncRNA | mRNA-ncRNA | -0.999953 | -1 |
| GPC5 | mRNA | ENST00000422749 | ncRNA | mRNA-ncRNA | -0.999953 | -1 |
| ADRB2 | mRNA | ENST00000422749 | ncRNA | mRNA-ncRNA | -0.999954 | -1 |
| ZNF215 | mRNA | ENST00000422749 | ncRNA | mRNA-ncRNA | -0.999960 | -1 |
| CHRFAM7A | mRNA | ENST00000422749 | ncRNA | mRNA-ncRNA | -0.999962 | -1 |
| GDA | mRNA | ENST00000422749 | ncRNA | mRNA-ncRNA | -0.999962 | -1 |
| CALD1 | mRNA | ENST00000422749 | ncRNA | mRNA-ncRNA | -0.999970 | -1 |
| ADAMTS6 | mRNA | ENST00000422749 | ncRNA | mRNA-ncRNA | -0.999972 | -1 |
| MLC1 | mRNA | ENST00000422749 | ncRNA | mRNA-ncRNA | -0.999973 | -1 |
| SRPX | mRNA | ENST00000422749 | ncRNA | mRNA-ncRNA | -0.999975 | -1 |
| NDN | mRNA | ENST00000422749 | ncRNA | mRNA-ncRNA | -0.999980 | -1 |
| ZCCHC11 | mRNA | ENST00000422749 | ncRNA | mRNA-ncRNA | -0.999980 | -1 |
| TMEM200A | mRNA | ENST00000422749 | ncRNA | mRNA-ncRNA | -0.999985 | -1 |
| KYNU | mRNA | ENST00000427113 | ncRNA | mRNA-ncRNA | 0.999959 | 1 |
| RNF145 | mRNA | ENST00000427113 | ncRNA | mRNA-ncRNA | -0.999969 | -1 |
| BSPRY | mRNA | ENST00000455354 | ncRNA | mRNA-ncRNA | 0.999960 | 1 |
| CXCL12 | mRNA | ENST00000455354 | ncRNA | mRNA-ncRNA | 0.999957 | 1 |
| SLC7A2 | mRNA | ENST00000455354 | ncRNA | mRNA-ncRNA | 0.999956 | 1 |
| CHRFAM7A | mRNA | ENST00000455354 | ncRNA | mRNA-ncRNA | -0.999951 | -1 |
| OSBPL3 | mRNA | ENST00000455354 | ncRNA | mRNA-ncRNA | -0.999963 | -1 |
| NDN | mRNA | ENST00000455354 | ncRNA | mRNA-ncRNA | -0.999972 | -1 |
| GPC5 | mRNA | ENST00000455354 | ncRNA | mRNA-ncRNA | -0.999974 | -1 |
| S1PR1 | mRNA | ENST00000455354 | ncRNA | mRNA-ncRNA | -0.999981 | -1 |
| LAMC3 | mRNA | ENST00000456355 | ncRNA | mRNA-ncRNA | 0.999994 | 1 |
| MCAM | mRNA | ENST00000456355 | ncRNA | mRNA-ncRNA | 0.999991 | 1 |
| GSDMD | mRNA | ENST00000456355 | ncRNA | mRNA-ncRNA | 0.999986 | 1 |
| FOSL1 | mRNA | ENST00000456355 | ncRNA | mRNA-ncRNA | 0.999984 | 1 |
| ST6GALNAC3 | mRNA | ENST00000456355 | ncRNA | mRNA-ncRNA | 0.999984 | 1 |
| SNRPN | mRNA | ENST00000456355 | ncRNA | mRNA-ncRNA | 0.999982 | 1 |
| TBX18 | mRNA | ENST00000456355 | ncRNA | mRNA-ncRNA | 0.999978 | 1 |
| LY6K | mRNA | ENST00000456355 | ncRNA | mRNA-ncRNA | 0.999974 | 1 |
| NNMT | mRNA | ENST00000456355 | ncRNA | mRNA-ncRNA | 0.999974 | 1 |
| MSN | mRNA | ENST00000456355 | ncRNA | mRNA-ncRNA | 0.999970 | 1 |
| SNURF | mRNA | ENST00000456355 | ncRNA | mRNA-ncRNA | 0.999966 | 1 |
| MSRB3 | mRNA | ENST00000456355 | ncRNA | mRNA-ncRNA | 0.999960 | 1 |
| TMEM200B | mRNA | ENST00000456355 | ncRNA | mRNA-ncRNA | 0.999954 | 1 |
| PRLR | mRNA | ENST00000456355 | ncRNA | mRNA-ncRNA | -0.999955 | -1 |
| SRPX | mRNA | ENST00000518943 | ncRNA | mRNA-ncRNA | 0.999983 | 1 |
| EVC | mRNA | ENST00000518943 | ncRNA | mRNA-ncRNA | 0.999979 | 1 |
| MLC1 | mRNA | ENST00000518943 | ncRNA | mRNA-ncRNA | 0.999968 | 1 |
| ZCCHC11 | mRNA | ENST00000518943 | ncRNA | mRNA-ncRNA | 0.999958 | 1 |
| CHRFAM7A | mRNA | ENST00000518943 | ncRNA | mRNA-ncRNA | 0.999958 | 1 |
| KRTAP2-3 | mRNA | ENST00000518943 | ncRNA | mRNA-ncRNA | 0.999951 | 1 |
| TMEM200A | mRNA | ENST00000518943 | ncRNA | mRNA-ncRNA | 0.999948 | 1 |
| TP53I11 | mRNA | ENST00000518943 | ncRNA | mRNA-ncRNA | -0.999964 | -1 |
| TSTD1 | mRNA | ENST00000518943 | ncRNA | mRNA-ncRNA | -0.999972 | -1 |
| TP53I11 | mRNA | ENST00000525867 | ncRNA | mRNA-ncRNA | 0.999948 | 1 |
| NDN | mRNA | ENST00000533697 | ncRNA | mRNA-ncRNA | 0.999995 | 1 |
| GPC5 | mRNA | ENST00000533697 | ncRNA | mRNA-ncRNA | 0.999994 | 1 |
| GDA | mRNA | ENST00000533697 | ncRNA | mRNA-ncRNA | 0.999992 | 1 |
| CALD1 | mRNA | ENST00000533697 | ncRNA | mRNA-ncRNA | 0.999992 | 1 |
| TMEM200A | mRNA | ENST00000533697 | ncRNA | mRNA-ncRNA | 0.999985 | 1 |
| ADRB2 | mRNA | ENST00000533697 | ncRNA | mRNA-ncRNA | 0.999984 | 1 |
| CHRFAM7A | mRNA | ENST00000533697 | ncRNA | mRNA-ncRNA | 0.999979 | 1 |
| UCHL1 | mRNA | ENST00000533697 | ncRNA | mRNA-ncRNA | 0.999979 | 1 |
| ADAMTS6 | mRNA | ENST00000533697 | ncRNA | mRNA-ncRNA | 0.999974 | 1 |
| ZNF215 | mRNA | ENST00000533697 | ncRNA | mRNA-ncRNA | 0.999974 | 1 |
| TMEM200B | mRNA | ENST00000533697 | ncRNA | mRNA-ncRNA | 0.999969 | 1 |
| SRPX | mRNA | ENST00000533697 | ncRNA | mRNA-ncRNA | 0.999967 | 1 |
| S1PR1 | mRNA | ENST00000533697 | ncRNA | mRNA-ncRNA | 0.999966 | 1 |
| PSG8 | mRNA | ENST00000533697 | ncRNA | mRNA-ncRNA | 0.999966 | 1 |
| RAB3B | mRNA | ENST00000533697 | ncRNA | mRNA-ncRNA | 0.999956 | 1 |
| TP53I11 | mRNA | ENST00000533697 | ncRNA | mRNA-ncRNA | -0.999958 | -1 |
| ARMT1 | mRNA | ENST00000551631 | ncRNA | mRNA-ncRNA | -0.999960 | -1 |
| MREG | mRNA | ENST00000551631 | ncRNA | mRNA-ncRNA | -0.999976 | -1 |
| MALL | mRNA | ENST00000585765 | ncRNA | mRNA-ncRNA | 0.999959 | 1 |
| PSMB8 | mRNA | ENST00000594783 | ncRNA | mRNA-ncRNA | 0.999981 | 1 |
| LAMC2 | mRNA | ENST00000594783 | ncRNA | mRNA-ncRNA | 0.999971 | 1 |
| IGFBP3 | mRNA | ENST00000594783 | ncRNA | mRNA-ncRNA | 0.999962 | 1 |
| SAMD3 | mRNA | ENST00000594783 | ncRNA | mRNA-ncRNA | 0.999954 | 1 |
| THSD1 | mRNA | ENST00000594783 | ncRNA | mRNA-ncRNA | 0.999953 | 1 |
| ADRB2 | mRNA | ENST00000594783 | ncRNA | mRNA-ncRNA | 0.999951 | 1 |
| EVC | mRNA | NONHSAT010369 | ncRNA | mRNA-ncRNA | 0.999961 | 1 |
| RTN1 | mRNA | NONHSAT033607 | ncRNA | mRNA-ncRNA | 0.999961 | 1 |
| PROS1 | mRNA | NONHSAT033607 | ncRNA | mRNA-ncRNA | 0.999953 | 1 |
| RGS9 | mRNA | NONHSAT033607 | ncRNA | mRNA-ncRNA | 0.999948 | 1 |
| IGFBP3 | mRNA | NONHSAT034295 | ncRNA | mRNA-ncRNA | 0.999984 | 1 |
| THSD1 | mRNA | NONHSAT034295 | ncRNA | mRNA-ncRNA | 0.999959 | 1 |
| SH3RF3 | mRNA | NONHSAT034295 | ncRNA | mRNA-ncRNA | 0.999955 | 1 |
| ABCC3 | mRNA | NONHSAT034295 | ncRNA | mRNA-ncRNA | -0.999977 | -1 |
| IRS1 | mRNA | NONHSAT044096 | ncRNA | mRNA-ncRNA | 0.999991 | 1 |
| CRYBG1 | mRNA | NONHSAT046604 | ncRNA | mRNA-ncRNA | 0.999984 | 1 |
| GNB2 | mRNA | NONHSAT046604 | ncRNA | mRNA-ncRNA | 0.999964 | 1 |
| NDN | mRNA | NONHSAT057283 | ncRNA | mRNA-ncRNA | 0.999989 | 1 |
| TMEM106A | mRNA | NONHSAT057283 | ncRNA | mRNA-ncRNA | 0.999986 | 1 |
| MLC1 | mRNA | NONHSAT057283 | ncRNA | mRNA-ncRNA | 0.999981 | 1 |
| S1PR1 | mRNA | NONHSAT057283 | ncRNA | mRNA-ncRNA | 0.999980 | 1 |
| ARHGAP40 | mRNA | NONHSAT057283 | ncRNA | mRNA-ncRNA | 0.999979 | 1 |
| ZCCHC11 | mRNA | NONHSAT057283 | ncRNA | mRNA-ncRNA | 0.999978 | 1 |
| PSG8 | mRNA | NONHSAT057283 | ncRNA | mRNA-ncRNA | 0.999971 | 1 |
| TMEM200A | mRNA | NONHSAT057283 | ncRNA | mRNA-ncRNA | 0.999963 | 1 |
| CHRFAM7A | mRNA | NONHSAT057283 | ncRNA | mRNA-ncRNA | 0.999962 | 1 |
| GDA | mRNA | NONHSAT057283 | ncRNA | mRNA-ncRNA | 0.999959 | 1 |
| CD274 | mRNA | NONHSAT057283 | ncRNA | mRNA-ncRNA | 0.999957 | 1 |
| SRPX | mRNA | NONHSAT057283 | ncRNA | mRNA-ncRNA | 0.999956 | 1 |
| CALD1 | mRNA | NONHSAT057283 | ncRNA | mRNA-ncRNA | 0.999954 | 1 |
| ADRB2 | mRNA | NONHSAT057283 | ncRNA | mRNA-ncRNA | 0.999951 | 1 |
| KRTAP2-3 | mRNA | NONHSAT057283 | ncRNA | mRNA-ncRNA | 0.999948 | 1 |
| BATF | mRNA | NONHSAT057283 | ncRNA | mRNA-ncRNA | -0.999951 | -1 |
| TP53I11 | mRNA | NONHSAT057283 | ncRNA | mRNA-ncRNA | -0.999961 | -1 |
| BSPRY | mRNA | NONHSAT057283 | ncRNA | mRNA-ncRNA | -0.999964 | -1 |
| MYOM3 | mRNA | NONHSAT072135 | ncRNA | mRNA-ncRNA | -0.999977 | -1 |
| PSG8 | mRNA | NONHSAT081899 | ncRNA | mRNA-ncRNA | 0.999989 | 1 |
| GDA | mRNA | NONHSAT081899 | ncRNA | mRNA-ncRNA | 0.999988 | 1 |
| NDN | mRNA | NONHSAT081899 | ncRNA | mRNA-ncRNA | 0.999987 | 1 |
| ADRB2 | mRNA | NONHSAT081899 | ncRNA | mRNA-ncRNA | 0.999984 | 1 |
| CALD1 | mRNA | NONHSAT081899 | ncRNA | mRNA-ncRNA | 0.999981 | 1 |
| GPC5 | mRNA | NONHSAT081899 | ncRNA | mRNA-ncRNA | 0.999975 | 1 |
| TMEM200A | mRNA | NONHSAT081899 | ncRNA | mRNA-ncRNA | 0.999971 | 1 |
| UCHL1 | mRNA | NONHSAT081899 | ncRNA | mRNA-ncRNA | 0.999962 | 1 |
| TMEM106A | mRNA | NONHSAT081899 | ncRNA | mRNA-ncRNA | 0.999958 | 1 |
| CHRFAM7A | mRNA | NONHSAT081899 | ncRNA | mRNA-ncRNA | 0.999956 | 1 |
| OSBPL3 | mRNA | NONHSAT081899 | ncRNA | mRNA-ncRNA | 0.999955 | 1 |
| ARHGAP40 | mRNA | NONHSAT081899 | ncRNA | mRNA-ncRNA | 0.999952 | 1 |
| STEAP1B | mRNA | NONHSAT081899 | ncRNA | mRNA-ncRNA | 0.999951 | 1 |
| ZCCHC11 | mRNA | NONHSAT081899 | ncRNA | mRNA-ncRNA | 0.999951 | 1 |
| S1PR1 | mRNA | NONHSAT081899 | ncRNA | mRNA-ncRNA | 0.999950 | 1 |
| BATF | mRNA | NONHSAT081899 | ncRNA | mRNA-ncRNA | -0.999948 | -1 |
| SLC7A2 | mRNA | NONHSAT081899 | ncRNA | mRNA-ncRNA | -0.999958 | -1 |
| CXCL12 | mRNA | NONHSAT082326 | ncRNA | mRNA-ncRNA | 0.999978 | 1 |
| IRS1 | mRNA | NONHSAT082353 | ncRNA | mRNA-ncRNA | 0.999957 | 1 |
| PKIB | mRNA | NONHSAT087654 | ncRNA | mRNA-ncRNA | 0.999965 | 1 |
| RAI14 | mRNA | NONHSAT087654 | ncRNA | mRNA-ncRNA | -0.999959 | -1 |
| PLEKHO2 | mRNA | NONHSAT087654 | ncRNA | mRNA-ncRNA | -0.999960 | -1 |
| AEBP1 | mRNA | NONHSAT090388 | ncRNA | mRNA-ncRNA | -0.999974 | -1 |
| CA12 | mRNA | NONHSAT097797 | ncRNA | mRNA-ncRNA | 0.999994 | 1 |
| BSPRY | mRNA | NONHSAT097797 | ncRNA | mRNA-ncRNA | 0.999986 | 1 |
| C5AR2 | mRNA | NONHSAT097797 | ncRNA | mRNA-ncRNA | 0.999978 | 1 |
| CDH1 | mRNA | NONHSAT097797 | ncRNA | mRNA-ncRNA | 0.999954 | 1 |
| NDN | mRNA | NONHSAT097797 | ncRNA | mRNA-ncRNA | -0.999949 | -1 |
| ZCCHC11 | mRNA | NONHSAT097797 | ncRNA | mRNA-ncRNA | -0.999963 | -1 |
| MLC1 | mRNA | NONHSAT097797 | ncRNA | mRNA-ncRNA | -0.999965 | -1 |
| SMC1B | mRNA | NONHSAT098643 | ncRNA | mRNA-ncRNA | 0.999982 | 1 |
| NNMT | mRNA | NONHSAT098643 | ncRNA | mRNA-ncRNA | 0.999978 | 1 |
| MSRB3 | mRNA | NONHSAT098643 | ncRNA | mRNA-ncRNA | 0.999970 | 1 |
| LAMC3 | mRNA | NONHSAT098643 | ncRNA | mRNA-ncRNA | 0.999969 | 1 |
| LY6K | mRNA | NONHSAT098643 | ncRNA | mRNA-ncRNA | 0.999964 | 1 |
| ST6GALNAC3 | mRNA | NONHSAT098643 | ncRNA | mRNA-ncRNA | 0.999962 | 1 |
| GSDMD | mRNA | NONHSAT098643 | ncRNA | mRNA-ncRNA | 0.999957 | 1 |
| SNURF | mRNA | NONHSAT098643 | ncRNA | mRNA-ncRNA | 0.999956 | 1 |
| FOSL1 | mRNA | NONHSAT098643 | ncRNA | mRNA-ncRNA | 0.999954 | 1 |
| MCAM | mRNA | NONHSAT098643 | ncRNA | mRNA-ncRNA | 0.999951 | 1 |
| TPD52L1 | mRNA | NONHSAT099026 | ncRNA | mRNA-ncRNA | -0.999975 | -1 |
| EVC | mRNA | NONHSAT134851 | ncRNA | mRNA-ncRNA | 0.999974 | 1 |
| SRPX | mRNA | NONHSAT134851 | ncRNA | mRNA-ncRNA | 0.999958 | 1 |
| TSTD1 | mRNA | NONHSAT134851 | ncRNA | mRNA-ncRNA | -0.999952 | -1 |
| HIPK1 | mRNA | NONHSAT139199 | ncRNA | mRNA-ncRNA | 0.999958 | 1 |
| RHOJ | mRNA | NONHSAT139199 | ncRNA | mRNA-ncRNA | -0.999949 | -1 |
| RAMP2 | mRNA | NONHSAT139199 | ncRNA | mRNA-ncRNA | -0.999964 | -1 |
| FMN2 | mRNA | NONHSAT139199 | ncRNA | mRNA-ncRNA | -0.999998 | -1 |
| RBFOX3 | mRNA | NONHSAT139214 | ncRNA | mRNA-ncRNA | 0.999981 | 1 |
| PRR15L | mRNA | NONHSAT139214 | ncRNA | mRNA-ncRNA | -0.999958 | -1 |
| ACKR3 | mRNA | NONHSAT139214 | ncRNA | mRNA-ncRNA | -0.999981 | -1 |
| CD274 | mRNA | NONHSAT144709 | ncRNA | mRNA-ncRNA | 0.999983 | 1 |
| ZCCHC11 | mRNA | NONHSAT144709 | ncRNA | mRNA-ncRNA | 0.999981 | 1 |
| SLC18B1 | mRNA | NONHSAT144709 | ncRNA | mRNA-ncRNA | 0.999973 | 1 |
| MLC1 | mRNA | NONHSAT144709 | ncRNA | mRNA-ncRNA | 0.999964 | 1 |
| TMEM106A | mRNA | NONHSAT144709 | ncRNA | mRNA-ncRNA | 0.999959 | 1 |
| OR2W3 | mRNA | NONHSAT144709 | ncRNA | mRNA-ncRNA | 0.999949 | 1 |
| BATF | mRNA | NONHSAT144709 | ncRNA | mRNA-ncRNA | -0.999970 | -1 |
| CLIC4 | mRNA | NR_001446.2 | ncRNA | mRNA-ncRNA | 0.999956 | 1 |
| ITGA4 | mRNA | NR_001446.2 | ncRNA | mRNA-ncRNA | 0.999949 | 1 |
| LYPD6B | mRNA | NR_001446.2 | ncRNA | mRNA-ncRNA | -0.999957 | -1 |
| DOLK | mRNA | NR_033655.1 | ncRNA | mRNA-ncRNA | 0.999976 | 1 |
| C5AR2 | mRNA | NR_033655.1 | ncRNA | mRNA-ncRNA | 0.999971 | 1 |
| CA12 | mRNA | NR_033655.1 | ncRNA | mRNA-ncRNA | 0.999966 | 1 |
| SH3GLB1 | mRNA | NR_033655.1 | ncRNA | mRNA-ncRNA | -0.999952 | -1 |
| HLA-DPB1 | mRNA | NR_036641.1 | ncRNA | mRNA-ncRNA | 0.999978 | 1 |
| AGPS | mRNA | NR_036641.1 | ncRNA | mRNA-ncRNA | 0.999976 | 1 |
| PDK3 | mRNA | NR_036641.1 | ncRNA | mRNA-ncRNA | -0.999990 | -1 |
| ACKR3 | mRNA | NR_036685.1 | ncRNA | mRNA-ncRNA | 0.999989 | 1 |
| PRR15L | mRNA | NR_036685.1 | ncRNA | mRNA-ncRNA | 0.999979 | 1 |
| THSD1 | mRNA | NR_036685.1 | ncRNA | mRNA-ncRNA | -0.999948 | -1 |
| RBFOX3 | mRNA | NR_036685.1 | ncRNA | mRNA-ncRNA | -0.999980 | -1 |
| ESRP2 | mRNA | NR_037910.1 | ncRNA | mRNA-ncRNA | 0.999993 | 1 |
| ADIRF | mRNA | NR_037910.1 | ncRNA | mRNA-ncRNA | 0.999982 | 1 |
| BEX1 | mRNA | NR_037910.1 | ncRNA | mRNA-ncRNA | -0.999978 | -1 |
| PSG8 | mRNA | NR_038340.1 | ncRNA | mRNA-ncRNA | 0.999982 | 1 |
| ARHGAP40 | mRNA | NR_038340.1 | ncRNA | mRNA-ncRNA | 0.999958 | 1 |
| RBFOX3 | mRNA | NR_038340.1 | ncRNA | mRNA-ncRNA | 0.999957 | 1 |
| CD274 | mRNA | NR_038340.1 | ncRNA | mRNA-ncRNA | 0.999953 | 1 |
| ETV5 | mRNA | NR_038340.1 | ncRNA | mRNA-ncRNA | 0.999952 | 1 |
| ACKR3 | mRNA | NR_038340.1 | ncRNA | mRNA-ncRNA | -0.999951 | -1 |
| BATF | mRNA | NR_038340.1 | ncRNA | mRNA-ncRNA | -0.999980 | -1 |
| DZIP1 | mRNA | NR_046308.1 | ncRNA | mRNA-ncRNA | 0.999960 | 1 |
| GDA | mRNA | NR_073179.1 | ncRNA | mRNA-ncRNA | 0.999979 | 1 |
| ADRB2 | mRNA | NR_073179.1 | ncRNA | mRNA-ncRNA | 0.999978 | 1 |
| GPC5 | mRNA | NR_073179.1 | ncRNA | mRNA-ncRNA | 0.999974 | 1 |
| PSG8 | mRNA | NR_073179.1 | ncRNA | mRNA-ncRNA | 0.999974 | 1 |
| UCHL1 | mRNA | NR_073179.1 | ncRNA | mRNA-ncRNA | 0.999971 | 1 |
| CALD1 | mRNA | NR_073179.1 | ncRNA | mRNA-ncRNA | 0.999968 | 1 |
| OSBPL3 | mRNA | NR_073179.1 | ncRNA | mRNA-ncRNA | 0.999965 | 1 |
| STEAP1B | mRNA | NR_073179.1 | ncRNA | mRNA-ncRNA | 0.999961 | 1 |
| MSN | mRNA | NR_073179.1 | ncRNA | mRNA-ncRNA | 0.999959 | 1 |
| PSMB8 | mRNA | NR_073179.1 | ncRNA | mRNA-ncRNA | 0.999959 | 1 |
| NDN | mRNA | NR_073179.1 | ncRNA | mRNA-ncRNA | 0.999957 | 1 |
| RNF217 | mRNA | NR_073179.1 | ncRNA | mRNA-ncRNA | 0.999955 | 1 |
| ETV5 | mRNA | NR_073179.1 | ncRNA | mRNA-ncRNA | 0.999950 | 1 |
| SLC7A2 | mRNA | NR_073179.1 | ncRNA | mRNA-ncRNA | -0.999957 | -1 |
| ANXA3 | mRNA | TCONS_00000744 | ncRNA | mRNA-ncRNA | 0.999961 | 1 |
| SMIM10 | mRNA | TCONS_00000744 | ncRNA | mRNA-ncRNA | 0.999958 | 1 |
| NDN | mRNA | TCONS_00017929 | ncRNA | mRNA-ncRNA | 0.999993 | 1 |
| PSG8 | mRNA | TCONS_00017929 | ncRNA | mRNA-ncRNA | 0.999984 | 1 |
| TMEM106A | mRNA | TCONS_00017929 | ncRNA | mRNA-ncRNA | 0.999981 | 1 |
| GDA | mRNA | TCONS_00017929 | ncRNA | mRNA-ncRNA | 0.999980 | 1 |
| ZCCHC11 | mRNA | TCONS_00017929 | ncRNA | mRNA-ncRNA | 0.999979 | 1 |
| TMEM200A | mRNA | TCONS_00017929 | ncRNA | mRNA-ncRNA | 0.999979 | 1 |
| CALD1 | mRNA | TCONS_00017929 | ncRNA | mRNA-ncRNA | 0.999975 | 1 |
| ADRB2 | mRNA | TCONS_00017929 | ncRNA | mRNA-ncRNA | 0.999975 | 1 |
| MLC1 | mRNA | TCONS_00017929 | ncRNA | mRNA-ncRNA | 0.999975 | 1 |
| ARHGAP40 | mRNA | TCONS_00017929 | ncRNA | mRNA-ncRNA | 0.999967 | 1 |
| CHRFAM7A | mRNA | TCONS_00017929 | ncRNA | mRNA-ncRNA | 0.999964 | 1 |
| S1PR1 | mRNA | TCONS_00017929 | ncRNA | mRNA-ncRNA | 0.999964 | 1 |
| GPC5 | mRNA | TCONS_00017929 | ncRNA | mRNA-ncRNA | 0.999959 | 1 |
| CD274 | mRNA | TCONS_00017929 | ncRNA | mRNA-ncRNA | 0.999957 | 1 |
| SRPX | mRNA | TCONS_00017929 | ncRNA | mRNA-ncRNA | 0.999956 | 1 |
| BSPRY | mRNA | TCONS_00017929 | ncRNA | mRNA-ncRNA | -0.999948 | -1 |
| BATF | mRNA | TCONS_00017929 | ncRNA | mRNA-ncRNA | -0.999962 | -1 |
| TP53I11 | mRNA | TCONS_00017929 | ncRNA | mRNA-ncRNA | -0.999963 | -1 |
| ZNF22 | mRNA | TCONS_00026825 | ncRNA | mRNA-ncRNA | 0.999969 | 1 |
| MXRA7 | mRNA | TCONS_00029159 | ncRNA | mRNA-ncRNA | 0.999967 | 1 |
| PPM1D | mRNA | TCONS_00029159 | ncRNA | mRNA-ncRNA | -0.999988 | -1 |
| IL1A | mRNA | TCONS_l2_00001295 | ncRNA | mRNA-ncRNA | 0.999966 | 1 |
| RBFOX3 | mRNA | TCONS_l2_00002667 | ncRNA | mRNA-ncRNA | 0.999978 | 1 |
| PSG8 | mRNA | TCONS_l2_00002667 | ncRNA | mRNA-ncRNA | 0.999976 | 1 |
| ARHGAP40 | mRNA | TCONS_l2_00002667 | ncRNA | mRNA-ncRNA | 0.999963 | 1 |
| ETV5 | mRNA | TCONS_l2_00002667 | ncRNA | mRNA-ncRNA | 0.999958 | 1 |
| BATF | mRNA | TCONS_l2_00002667 | ncRNA | mRNA-ncRNA | -0.999952 | -1 |
| SLC7A2 | mRNA | TCONS_l2_00002667 | ncRNA | mRNA-ncRNA | -0.999959 | -1 |
| ACKR3 | mRNA | TCONS_l2_00002667 | ncRNA | mRNA-ncRNA | -0.999963 | -1 |
| FOSL1 | mRNA | TCONS_l2_00004346 | ncRNA | mRNA-ncRNA | 0.999961 | 1 |
| SNURF | mRNA | TCONS_l2_00004346 | ncRNA | mRNA-ncRNA | 0.999954 | 1 |
| NNMT | mRNA | TCONS_l2_00004346 | ncRNA | mRNA-ncRNA | 0.999952 | 1 |
| MFSD1 | mRNA | TCONS_l2_00004346 | ncRNA | mRNA-ncRNA | -0.999959 | -1 |
| RSPO4 | mRNA | TCONS_l2_00008290 | ncRNA | mRNA-ncRNA | 0.999963 | 1 |
| LIMS2 | mRNA | TCONS_l2_00008290 | ncRNA | mRNA-ncRNA | 0.999948 | 1 |
| SNURF | mRNA | TCONS_l2_00010132 | ncRNA | mRNA-ncRNA | 0.999984 | 1 |
| DGLUCY | mRNA | TCONS_l2_00010132 | ncRNA | mRNA-ncRNA | 0.999981 | 1 |
| TPM2 | mRNA | TCONS_l2_00010132 | ncRNA | mRNA-ncRNA | 0.999976 | 1 |
| NNMT | mRNA | TCONS_l2_00010132 | ncRNA | mRNA-ncRNA | 0.999955 | 1 |
| MSRB3 | mRNA | TCONS_l2_00010132 | ncRNA | mRNA-ncRNA | 0.999948 | 1 |
| GRHL2 | mRNA | TCONS_l2_00011199 | ncRNA | mRNA-ncRNA | 0.999951 | 1 |
| RNF152 | mRNA | TCONS_l2_00011199 | ncRNA | mRNA-ncRNA | 0.999949 | 1 |
| ZNF22 | mRNA | TCONS_l2_00014790 | ncRNA | mRNA-ncRNA | 0.999960 | 1 |
| UCHL1 | mRNA | TCONS_l2_00014790 | ncRNA | mRNA-ncRNA | 0.999956 | 1 |
| BTG3 | mRNA | TCONS_l2_00016247 | ncRNA | mRNA-ncRNA | 0.999978 | 1 |
| DZIP1 | mRNA | TCONS_l2_00016247 | ncRNA | mRNA-ncRNA | 0.999959 | 1 |
| NT5E | mRNA | TCONS_l2_00016247 | ncRNA | mRNA-ncRNA | 0.999953 | 1 |
| CRYBG1 | mRNA | TCONS_l2_00016247 | ncRNA | mRNA-ncRNA | -0.999975 | -1 |
| STARD10 | mRNA | TCONS_l2_00023638 | ncRNA | mRNA-ncRNA | 0.999973 | 1 |
| ANXA6 | mRNA | TCONS_l2_00026243 | ncRNA | mRNA-ncRNA | -0.999964 | -1 |
| ZNF717 | mRNA | TCONS_l2_00026243 | ncRNA | mRNA-ncRNA | -0.999974 | -1 |
| TSPO | mRNA | TCONS_l2_00026243 | ncRNA | mRNA-ncRNA | -0.999978 | -1 |
| ANKRD1 | mRNA | XR_133570.3 | ncRNA | mRNA-ncRNA | -0.999955 | -1 |
| CALCOCO1 | mRNA | XR_133570.3 | ncRNA | mRNA-ncRNA | -0.999982 | -1 |
| SLC18B1 | mRNA | XR_171924.2 | ncRNA | mRNA-ncRNA | 0.999990 | 1 |
| KCNJ18 | mRNA | XR_171924.2 | ncRNA | mRNA-ncRNA | 0.999987 | 1 |
| FGFBP1 | mRNA | XR_171924.2 | ncRNA | mRNA-ncRNA | 0.999986 | 1 |
| SGCE | mRNA | XR_171924.2 | ncRNA | mRNA-ncRNA | 0.999974 | 1 |
| FGF5 | mRNA | XR_171924.2 | ncRNA | mRNA-ncRNA | 0.999972 | 1 |
| ANKLE1 | mRNA | XR_171924.2 | ncRNA | mRNA-ncRNA | 0.999971 | 1 |
| CD274 | mRNA | XR_171924.2 | ncRNA | mRNA-ncRNA | 0.999963 | 1 |
| MAEA | mRNA | XR_171924.2 | ncRNA | mRNA-ncRNA | 0.999961 | 1 |
| EVC | mRNA | XR_241036.1 | ncRNA | mRNA-ncRNA | 0.999969 | 1 |
| ADAMTS6 | mRNA | XR_241036.1 | ncRNA | mRNA-ncRNA | 0.999957 | 1 |
| RYR1 | mRNA | XR_241036.1 | ncRNA | mRNA-ncRNA | 0.999948 | 1 |
| RSPO4 | mRNA | XR_241105.1 | ncRNA | mRNA-ncRNA | -0.999956 | -1 |
| ST6GALNAC3 | mRNA | XR_241105.1 | ncRNA | mRNA-ncRNA | -0.999964 | -1 |
| LIMS2 | mRNA | XR_241105.1 | ncRNA | mRNA-ncRNA | -0.999966 | -1 |
| FOSL1 | mRNA | XR_241105.1 | ncRNA | mRNA-ncRNA | -0.999971 | -1 |
| ZNF22 | mRNA | XR_241105.1 | ncRNA | mRNA-ncRNA | -0.999984 | -1 |
| RTN1 | mRNA | XR_241491.1 | ncRNA | mRNA-ncRNA | 0.999987 | 1 |
| RGS9 | mRNA | XR_241491.1 | ncRNA | mRNA-ncRNA | 0.999961 | 1 |
| MMP1 | mRNA | XR_241491.1 | ncRNA | mRNA-ncRNA | 0.999955 | 1 |
| RPRML | mRNA | XR_242373.1 | ncRNA | mRNA-ncRNA | 0.999956 | 1 |
| ZNF22 | mRNA | XR_242373.1 | ncRNA | mRNA-ncRNA | 0.999954 | 1 |
| IRS1 | mRNA | XR_243111.1 | ncRNA | mRNA-ncRNA | 0.999960 | 1 |
| HIST1H2BK | mRNA | XR_243111.1 | ncRNA | mRNA-ncRNA | 0.999956 | 1 |
| ZFAND4 | mRNA | XR_243133.1 | ncRNA | mRNA-ncRNA | 0.999956 | 1 |
| FXYD5 | mRNA | XR_243133.1 | ncRNA | mRNA-ncRNA | 0.999951 | 1 |
| BTG3 | mRNA | XR_243402.1 | ncRNA | mRNA-ncRNA | 0.999961 | 1 |
| PDK3 | mRNA | XR_243402.1 | ncRNA | mRNA-ncRNA | -0.999962 | -1 |
| AGR2 | mRNA | XR_243500.1 | ncRNA | mRNA-ncRNA | 0.999970 | 1 |
| IDH1 | mRNA | XR_243500.1 | ncRNA | mRNA-ncRNA | 0.999953 | 1 |
| S1PR1 | mRNA | XR_245098.1 | ncRNA | mRNA-ncRNA | 0.999980 | 1 |
| FOXG1 | mRNA | XR_245098.1 | ncRNA | mRNA-ncRNA | 0.999978 | 1 |
| MLC1 | mRNA | XR_245098.1 | ncRNA | mRNA-ncRNA | 0.999975 | 1 |
| ZCCHC11 | mRNA | XR_245098.1 | ncRNA | mRNA-ncRNA | 0.999958 | 1 |
| CDC42EP3 | mRNA | XR_245098.1 | ncRNA | mRNA-ncRNA | 0.999955 | 1 |
| NDN | mRNA | XR_245098.1 | ncRNA | mRNA-ncRNA | 0.999953 | 1 |
| SRPX | mRNA | XR_245098.1 | ncRNA | mRNA-ncRNA | 0.999953 | 1 |
| KRTAP2-3 | mRNA | XR_245098.1 | ncRNA | mRNA-ncRNA | 0.999952 | 1 |
| TMEM106A | mRNA | XR_245098.1 | ncRNA | mRNA-ncRNA | 0.999952 | 1 |
| TSTD1 | mRNA | XR_245098.1 | ncRNA | mRNA-ncRNA | -0.999970 | -1 |
| BSPRY | mRNA | XR_245098.1 | ncRNA | mRNA-ncRNA | -0.999986 | -1 |
| NRCAM | mRNA | XR_245663.1 | ncRNA | mRNA-ncRNA | 0.999973 | 1 |
| TUBD1 | mRNA | XR_245663.1 | ncRNA | mRNA-ncRNA | 0.999960 | 1 |
| AEBP1 | mRNA | XR_245663.1 | ncRNA | mRNA-ncRNA | -0.999962 | -1 |
| LONRF3 | mRNA | XR_246765.1 | ncRNA | mRNA-ncRNA | 0.999986 | 1 |
| SH3RF3 | mRNA | XR_246765.1 | ncRNA | mRNA-ncRNA | 0.999981 | 1 |
| LRCH2 | mRNA | XR_246765.1 | ncRNA | mRNA-ncRNA | 0.999976 | 1 |
| PDPN | mRNA | XR_246765.1 | ncRNA | mRNA-ncRNA | 0.999966 | 1 |
| RAB38 | mRNA | XR_246765.1 | ncRNA | mRNA-ncRNA | 0.999964 | 1 |
| IGFBP3 | mRNA | XR_246765.1 | ncRNA | mRNA-ncRNA | 0.999962 | 1 |
| ABCC3 | mRNA | XR_246765.1 | ncRNA | mRNA-ncRNA | -0.999965 | -1 |
| SPDL1 | mRNA | XR_247172.1 | ncRNA | mRNA-ncRNA | 0.999967 | 1 |
| USP32 | mRNA | XR_247172.1 | ncRNA | mRNA-ncRNA | -0.999981 | -1 |
| RFLNB | mRNA | APOBEC3G | mRNA | mRNA-mRNA | 0.999999 | 1 |
| TBX18 | mRNA | SNRPN | mRNA | mRNA-mRNA | 0.999999 | 1 |
| GDA | mRNA | ADRB2 | mRNA | mRNA-mRNA | 0.999998 | 1 |
| LOC100128988 | mRNA | HIST1H2BK | mRNA | mRNA-mRNA | 0.999998 | 1 |
| GDA | mRNA | CALD1 | mRNA | mRNA-mRNA | 0.999998 | 1 |
| ZCCHC11 | mRNA | MLC1 | mRNA | mRNA-mRNA | 0.999997 | 1 |
| RSPO4 | mRNA | LIMS2 | mRNA | mRNA-mRNA | 0.999996 | 1 |
| CALD1 | mRNA | ADRB2 | mRNA | mRNA-mRNA | 0.999996 | 1 |
| FGFBP1 | mRNA | FGF5 | mRNA | mRNA-mRNA | 0.999996 | 1 |
| POF1B | mRNA | CXXC5 | mRNA | mRNA-mRNA | 0.999995 | 1 |
| ELAVL2 | mRNA | CT45A5 | mRNA | mRNA-mRNA | 0.999995 | 1 |
| SPAG16 | mRNA | FGD1 | mRNA | mRNA-mRNA | 0.999995 | 1 |
| MCAM | mRNA | LAMC3 | mRNA | mRNA-mRNA | 0.999995 | 1 |
| HLA-A | mRNA | CNN1 | mRNA | mRNA-mRNA | 0.999995 | 1 |
| MSN | mRNA | GSDMD | mRNA | mRNA-mRNA | 0.999994 | 1 |
| TMEM200A | mRNA | CALD1 | mRNA | mRNA-mRNA | 0.999994 | 1 |
| KCNJ18 | mRNA | ANKLE1 | mRNA | mRNA-mRNA | 0.999994 | 1 |
| UCHL1 | mRNA | TMEM200B | mRNA | mRNA-mRNA | 0.999993 | 1 |
| KCNJ18 | mRNA | FGFBP1 | mRNA | mRNA-mRNA | 0.999993 | 1 |
| ZNF597 | mRNA | KDF1 | mRNA | mRNA-mRNA | 0.999992 | 1 |
| ZFPM2 | mRNA | G0S2 | mRNA | mRNA-mRNA | 0.999992 | 1 |
| SRPX | mRNA | CHRFAM7A | mRNA | mRNA-mRNA | 0.999992 | 1 |
| MCAM | mRNA | GSDMD | mRNA | mRNA-mRNA | 0.999992 | 1 |
| TMEM200A | mRNA | ADAMTS6 | mRNA | mRNA-mRNA | 0.999991 | 1 |
| SNURF | mRNA | DGLUCY | mRNA | mRNA-mRNA | 0.999991 | 1 |
| SH3RF3 | mRNA | PDPN | mRNA | mRNA-mRNA | 0.999991 | 1 |
| LY6K | mRNA | GSDMD | mRNA | mRNA-mRNA | 0.999991 | 1 |
| SNRPN | mRNA | MCAM | mRNA | mRNA-mRNA | 0.999991 | 1 |
| MSRB3 | mRNA | LY6K | mRNA | mRNA-mRNA | 0.999991 | 1 |
| ESRP2 | mRNA | ADIRF | mRNA | mRNA-mRNA | 0.999991 | 1 |
| NNMT | mRNA | LY6K | mRNA | mRNA-mRNA | 0.999991 | 1 |
| ST6GALNAC3 | mRNA | LAMC3 | mRNA | mRNA-mRNA | 0.999990 | 1 |
| SH3PXD2A | mRNA | LRRN4 | mRNA | mRNA-mRNA | 0.999990 | 1 |
| TBX18 | mRNA | MCAM | mRNA | mRNA-mRNA | 0.999990 | 1 |
| CDC42EP3 | mRNA | ANKLE1 | mRNA | mRNA-mRNA | 0.999989 | 1 |
| SNURF | mRNA | NNMT | mRNA | mRNA-mRNA | 0.999989 | 1 |
| MSN | mRNA | MCAM | mRNA | mRNA-mRNA | 0.999989 | 1 |
| NNMT | mRNA | MSRB3 | mRNA | mRNA-mRNA | 0.999989 | 1 |
| TMEM200A | mRNA | GDA | mRNA | mRNA-mRNA | 0.999989 | 1 |
| TMEM200A | mRNA | RAB3B | mRNA | mRNA-mRNA | 0.999988 | 1 |
| TMEM106A | mRNA | MLC1 | mRNA | mRNA-mRNA | 0.999988 | 1 |
| TMEM200A | mRNA | NDN | mRNA | mRNA-mRNA | 0.999988 | 1 |
| LAMC3 | mRNA | GSDMD | mRNA | mRNA-mRNA | 0.999987 | 1 |
| TMEM200A | mRNA | CHRFAM7A | mRNA | mRNA-mRNA | 0.999987 | 1 |
| ZCCHC11 | mRNA | TMEM106A | mRNA | mRNA-mRNA | 0.999987 | 1 |
| GPX1 | mRNA | CST6 | mRNA | mRNA-mRNA | 0.999987 | 1 |
| CA12 | mRNA | C5AR2 | mRNA | mRNA-mRNA | 0.999987 | 1 |
| NDN | mRNA | CHRFAM7A | mRNA | mRNA-mRNA | 0.999986 | 1 |
| FGFBP1 | mRNA | ANKLE1 | mRNA | mRNA-mRNA | 0.999986 | 1 |
| CALD1 | mRNA | ADAMTS6 | mRNA | mRNA-mRNA | 0.999986 | 1 |
| SNURF | mRNA | MSRB3 | mRNA | mRNA-mRNA | 0.999986 | 1 |
| NDN | mRNA | GDA | mRNA | mRNA-mRNA | 0.999986 | 1 |
| PTX3 | mRNA | CREB5 | mRNA | mRNA-mRNA | 0.999986 | 1 |
| UCHL1 | mRNA | GPC5 | mRNA | mRNA-mRNA | 0.999986 | 1 |
| MAEA | mRNA | KCNJ18 | mRNA | mRNA-mRNA | 0.999986 | 1 |
| SLC18B1 | mRNA | CD274 | mRNA | mRNA-mRNA | 0.999986 | 1 |
| TBX18 | mRNA | LAYN | mRNA | mRNA-mRNA | 0.999985 | 1 |
| TMEM200A | mRNA | ADRB2 | mRNA | mRNA-mRNA | 0.999985 | 1 |
| UCHL1 | mRNA | GDA | mRNA | mRNA-mRNA | 0.999985 | 1 |
| LIMS2 | mRNA | CSPG4 | mRNA | mRNA-mRNA | 0.999985 | 1 |
| NDN | mRNA | CALD1 | mRNA | mRNA-mRNA | 0.999985 | 1 |
| CHRFAM7A | mRNA | ADAMTS6 | mRNA | mRNA-mRNA | 0.999985 | 1 |
| SRPX | mRNA | ADAMTS6 | mRNA | mRNA-mRNA | 0.999985 | 1 |
| ZNF215 | mRNA | ADAMTS6 | mRNA | mRNA-mRNA | 0.999985 | 1 |
| RHOJ | mRNA | RAMP2 | mRNA | mRNA-mRNA | 0.999985 | 1 |
| SNURF | mRNA | LY6K | mRNA | mRNA-mRNA | 0.999985 | 1 |
| MAEA | mRNA | ANKLE1 | mRNA | mRNA-mRNA | 0.999984 | 1 |
| MAFB | mRNA | AGR3 | mRNA | mRNA-mRNA | 0.999984 | 1 |
| SNRPN | mRNA | FOSL1 | mRNA | mRNA-mRNA | 0.999984 | 1 |
| PDPN | mRNA | CST6 | mRNA | mRNA-mRNA | 0.999984 | 1 |
| PSG8 | mRNA | ADRB2 | mRNA | mRNA-mRNA | 0.999984 | 1 |
| TMEM200A | mRNA | SRPX | mRNA | mRNA-mRNA | 0.999984 | 1 |
| GPC5 | mRNA | CALD1 | mRNA | mRNA-mRNA | 0.999984 | 1 |
| RAB3B | mRNA | ADAMTS6 | mRNA | mRNA-mRNA | 0.999984 | 1 |
| GPC5 | mRNA | GDA | mRNA | mRNA-mRNA | 0.999984 | 1 |
| LOX | mRNA | FABP5 | mRNA | mRNA-mRNA | 0.999983 | 1 |
| RAB3B | mRNA | CALD1 | mRNA | mRNA-mRNA | 0.999983 | 1 |
| SNRPN | mRNA | LAYN | mRNA | mRNA-mRNA | 0.999983 | 1 |
| SAMD3 | mRNA | CST6 | mRNA | mRNA-mRNA | 0.999983 | 1 |
| ZFAND4 | mRNA | PTX3 | mRNA | mRNA-mRNA | 0.999983 | 1 |
| LRCH2 | mRNA | GAREM2 | mRNA | mRNA-mRNA | 0.999983 | 1 |
| SH3RF3 | mRNA | RAB38 | mRNA | mRNA-mRNA | 0.999983 | 1 |
| LAMA1 | mRNA | IL6 | mRNA | mRNA-mRNA | 0.999982 | 1 |
| DZIP1 | mRNA | BTG3 | mRNA | mRNA-mRNA | 0.999982 | 1 |
| TMEM200B | mRNA | GPC5 | mRNA | mRNA-mRNA | 0.999982 | 1 |
| UCHL1 | mRNA | CALD1 | mRNA | mRNA-mRNA | 0.999982 | 1 |
| MLC1 | mRNA | KRTAP2-3 | mRNA | mRNA-mRNA | 0.999982 | 1 |
| ZNF215 | mRNA | GPC5 | mRNA | mRNA-mRNA | 0.999982 | 1 |
| KCNJ18 | mRNA | FGF5 | mRNA | mRNA-mRNA | 0.999982 | 1 |
| ZFAND4 | mRNA | CREB5 | mRNA | mRNA-mRNA | 0.999982 | 1 |
| S1PR1 | mRNA | NDN | mRNA | mRNA-mRNA | 0.999982 | 1 |
| CHRFAM7A | mRNA | CALD1 | mRNA | mRNA-mRNA | 0.999981 | 1 |
| TBX18 | mRNA | GSDMD | mRNA | mRNA-mRNA | 0.999981 | 1 |
| ST6GALNAC3 | mRNA | FOSL1 | mRNA | mRNA-mRNA | 0.999981 | 1 |
| SNRPN | mRNA | GSDMD | mRNA | mRNA-mRNA | 0.999981 | 1 |
| FGD1 | mRNA | FABP5 | mRNA | mRNA-mRNA | 0.999981 | 1 |
| ZCCHC11 | mRNA | CD274 | mRNA | mRNA-mRNA | 0.999981 | 1 |
| PSG8 | mRNA | GDA | mRNA | mRNA-mRNA | 0.999981 | 1 |
| MSN | mRNA | LAYN | mRNA | mRNA-mRNA | 0.999980 | 1 |
| UCHL1 | mRNA | ADRB2 | mRNA | mRNA-mRNA | 0.999980 | 1 |
| RAB3B | mRNA | ADRB2 | mRNA | mRNA-mRNA | 0.999980 | 1 |
| MSN | mRNA | LY6K | mRNA | mRNA-mRNA | 0.999980 | 1 |
| RASIP1 | mRNA | LHFPL6 | mRNA | mRNA-mRNA | 0.999980 | 1 |
| MMP1 | mRNA | DCBLD2 | mRNA | mRNA-mRNA | 0.999980 | 1 |
| MCAM | mRNA | FOSL1 | mRNA | mRNA-mRNA | 0.999980 | 1 |
| TUBB6 | mRNA | RGS9 | mRNA | mRNA-mRNA | 0.999980 | 1 |
| MSN | mRNA | LAMC3 | mRNA | mRNA-mRNA | 0.999979 | 1 |
| TMEM200B | mRNA | LAMC3 | mRNA | mRNA-mRNA | 0.999979 | 1 |
| TBX18 | mRNA | MSN | mRNA | mRNA-mRNA | 0.999979 | 1 |
| FGF5 | mRNA | ANKLE1 | mRNA | mRNA-mRNA | 0.999979 | 1 |
| LSP1 | mRNA | AJUBA | mRNA | mRNA-mRNA | 0.999979 | 1 |
| SLC18B1 | mRNA | KCNJ18 | mRNA | mRNA-mRNA | 0.999979 | 1 |
| ST6GALNAC3 | mRNA | MCAM | mRNA | mRNA-mRNA | 0.999979 | 1 |
| SNRPN | mRNA | MSN | mRNA | mRNA-mRNA | 0.999979 | 1 |
| SRPX | mRNA | NDN | mRNA | mRNA-mRNA | 0.999978 | 1 |
| ZNF215 | mRNA | CALD1 | mRNA | mRNA-mRNA | 0.999978 | 1 |
| LAMC3 | mRNA | FOSL1 | mRNA | mRNA-mRNA | 0.999978 | 1 |
| NDN | mRNA | ADRB2 | mRNA | mRNA-mRNA | 0.999978 | 1 |
| MAEA | mRNA | CDC42EP3 | mRNA | mRNA-mRNA | 0.999978 | 1 |
| NNMT | mRNA | GSDMD | mRNA | mRNA-mRNA | 0.999978 | 1 |
| FLG-AS1 | mRNA | CD109 | mRNA | mRNA-mRNA | 0.999978 | 1 |
| NDN | mRNA | GPC5 | mRNA | mRNA-mRNA | 0.999978 | 1 |
| ZNF215 | mRNA | TMEM200B | mRNA | mRNA-mRNA | 0.999977 | 1 |
| FGF5 | mRNA | CD274 | mRNA | mRNA-mRNA | 0.999977 | 1 |
| GDA | mRNA | CHRFAM7A | mRNA | mRNA-mRNA | 0.999977 | 1 |
| TMEM106A | mRNA | KRTAP2-3 | mRNA | mRNA-mRNA | 0.999977 | 1 |
| STEAP1B | mRNA | PSG8 | mRNA | mRNA-mRNA | 0.999977 | 1 |
| PSMB8 | mRNA | ADRB2 | mRNA | mRNA-mRNA | 0.999977 | 1 |
| SLC18B1 | mRNA | FGFBP1 | mRNA | mRNA-mRNA | 0.999977 | 1 |
| PRTFDC1 | mRNA | MLLT11 | mRNA | mRNA-mRNA | 0.999977 | 1 |
| SAMD3 | mRNA | IGFBP3 | mRNA | mRNA-mRNA | 0.999977 | 1 |
| SNRPN | mRNA | LAMC3 | mRNA | mRNA-mRNA | 0.999977 | 1 |
| RAB3B | mRNA | GDA | mRNA | mRNA-mRNA | 0.999977 | 1 |
| SGCE | mRNA | PLAG1 | mRNA | mRNA-mRNA | 0.999977 | 1 |
| KRTAP2-3 | mRNA | ANKLE1 | mRNA | mRNA-mRNA | 0.999977 | 1 |
| SPAG16 | mRNA | MICU3 | mRNA | mRNA-mRNA | 0.999977 | 1 |
| LHFPL6 | mRNA | BTG3 | mRNA | mRNA-mRNA | 0.999977 | 1 |
| TMEM200B | mRNA | ST6GALNAC3 | mRNA | mRNA-mRNA | 0.999976 | 1 |
| MAEA | mRNA | KRTAP2-3 | mRNA | mRNA-mRNA | 0.999976 | 1 |
| RSPO4 | mRNA | CSPG4 | mRNA | mRNA-mRNA | 0.999976 | 1 |
| TBX18 | mRNA | FOSL1 | mRNA | mRNA-mRNA | 0.999976 | 1 |
| TMEM200B | mRNA | CALD1 | mRNA | mRNA-mRNA | 0.999976 | 1 |
| MLC1 | mRNA | CD274 | mRNA | mRNA-mRNA | 0.999976 | 1 |
| SH3RF3 | mRNA | IGFBP3 | mRNA | mRNA-mRNA | 0.999976 | 1 |
| PDPN | mRNA | GPX1 | mRNA | mRNA-mRNA | 0.999975 | 1 |
| SAMD3 | mRNA | RAB38 | mRNA | mRNA-mRNA | 0.999975 | 1 |
| KRTAP2-3 | mRNA | CDC42EP3 | mRNA | mRNA-mRNA | 0.999975 | 1 |
| PSMB8 | mRNA | MSN | mRNA | mRNA-mRNA | 0.999975 | 1 |
| GDA | mRNA | ADAMTS6 | mRNA | mRNA-mRNA | 0.999975 | 1 |
| LAMTOR5 | mRNA | ESRP1 | mRNA | mRNA-mRNA | 0.999975 | 1 |
| KYNU | mRNA | IRX2 | mRNA | mRNA-mRNA | 0.999975 | 1 |
| NNMT | mRNA | DGLUCY | mRNA | mRNA-mRNA | 0.999975 | 1 |
| ESPNL | mRNA | COTL1 | mRNA | mRNA-mRNA | 0.999975 | 1 |
| CA12 | mRNA | BSPRY | mRNA | mRNA-mRNA | 0.999975 | 1 |
| DDR1 | mRNA | BSCL2 | mRNA | mRNA-mRNA | 0.999974 | 1 |
| TMEM106A | mRNA | CD274 | mRNA | mRNA-mRNA | 0.999974 | 1 |
| S1PR1 | mRNA | CHRFAM7A | mRNA | mRNA-mRNA | 0.999974 | 1 |
| RET | mRNA | GFRA1 | mRNA | mRNA-mRNA | 0.999974 | 1 |
| LAYN | mRNA | GSDMD | mRNA | mRNA-mRNA | 0.999974 | 1 |
| RAB3B | mRNA | CHRFAM7A | mRNA | mRNA-mRNA | 0.999974 | 1 |
| GPC5 | mRNA | ADRB2 | mRNA | mRNA-mRNA | 0.999973 | 1 |
| ZSCAN12 | mRNA | RPRML | mRNA | mRNA-mRNA | 0.999973 | 1 |
| MICU3 | mRNA | FGD1 | mRNA | mRNA-mRNA | 0.999973 | 1 |
| SLC18B1 | mRNA | FGF5 | mRNA | mRNA-mRNA | 0.999973 | 1 |
| MSRB3 | mRNA | DGLUCY | mRNA | mRNA-mRNA | 0.999973 | 1 |
| TMEM45B | mRNA | MAFB | mRNA | mRNA-mRNA | 0.999973 | 1 |
| TSPO | mRNA | ANXA6 | mRNA | mRNA-mRNA | 0.999973 | 1 |
| TBX18 | mRNA | LAMC3 | mRNA | mRNA-mRNA | 0.999973 | 1 |
| SLC18B1 | mRNA | ANKLE1 | mRNA | mRNA-mRNA | 0.999973 | 1 |
| TPM2 | mRNA | DGLUCY | mRNA | mRNA-mRNA | 0.999973 | 1 |
| SPAG16 | mRNA | GAREM2 | mRNA | mRNA-mRNA | 0.999973 | 1 |
| STEAP1B | mRNA | ADRB2 | mRNA | mRNA-mRNA | 0.999973 | 1 |
| TMEM200B | mRNA | GDA | mRNA | mRNA-mRNA | 0.999972 | 1 |
| TMEM106A | mRNA | NDN | mRNA | mRNA-mRNA | 0.999972 | 1 |
| KCNJ18 | mRNA | CDC42EP3 | mRNA | mRNA-mRNA | 0.999972 | 1 |
| DZIP1 | mRNA | CT45A5 | mRNA | mRNA-mRNA | 0.999972 | 1 |
| ZCCHC11 | mRNA | KRTAP2-3 | mRNA | mRNA-mRNA | 0.999972 | 1 |
| PTPN7 | mRNA | CDIP1 | mRNA | mRNA-mRNA | 0.999972 | 1 |
| LRRN4 | mRNA | CD109 | mRNA | mRNA-mRNA | 0.999972 | 1 |
| RPS6KB1 | mRNA | MAFB | mRNA | mRNA-mRNA | 0.999972 | 1 |
| PLAG1 | mRNA | MAEA | mRNA | mRNA-mRNA | 0.999972 | 1 |
| MYL9 | mRNA | IL1A | mRNA | mRNA-mRNA | 0.999972 | 1 |
| CD274 | mRNA | ANKLE1 | mRNA | mRNA-mRNA | 0.999972 | 1 |
| TMEM200B | mRNA | ADAMTS6 | mRNA | mRNA-mRNA | 0.999972 | 1 |
| PSG8 | mRNA | NDN | mRNA | mRNA-mRNA | 0.999971 | 1 |
| SNURF | mRNA | GSDMD | mRNA | mRNA-mRNA | 0.999971 | 1 |
| NPHP1 | mRNA | GOLGA8A | mRNA | mRNA-mRNA | 0.999971 | 1 |
| TMEM106A | mRNA | PSG8 | mRNA | mRNA-mRNA | 0.999971 | 1 |
| RAB3B | mRNA | OR2W3 | mRNA | mRNA-mRNA | 0.999971 | 1 |
| RAB38 | mRNA | PDPN | mRNA | mRNA-mRNA | 0.999971 | 1 |
| WDR66 | mRNA | DCBLD2 | mRNA | mRNA-mRNA | 0.999971 | 1 |
| SRPX | mRNA | S1PR1 | mRNA | mRNA-mRNA | 0.999970 | 1 |
| ZNF215 | mRNA | TMEM200A | mRNA | mRNA-mRNA | 0.999970 | 1 |
| RAMP2 | mRNA | FMN2 | mRNA | mRNA-mRNA | 0.999970 | 1 |
| ZCCHC11 | mRNA | TMEM200A | mRNA | mRNA-mRNA | 0.999970 | 1 |
| LY6K | mRNA | DGLUCY | mRNA | mRNA-mRNA | 0.999970 | 1 |
| NDN | mRNA | ADAMTS6 | mRNA | mRNA-mRNA | 0.999970 | 1 |
| UCHL1 | mRNA | CHRFAM7A | mRNA | mRNA-mRNA | 0.999970 | 1 |
| ST6GALNAC3 | mRNA | LIMS2 | mRNA | mRNA-mRNA | 0.999970 | 1 |
| PSMB8 | mRNA | GSDMD | mRNA | mRNA-mRNA | 0.999970 | 1 |
| FGFBP1 | mRNA | CD274 | mRNA | mRNA-mRNA | 0.999970 | 1 |
| PSG8 | mRNA | CALD1 | mRNA | mRNA-mRNA | 0.999970 | 1 |
| MCAM | mRNA | LY6K | mRNA | mRNA-mRNA | 0.999970 | 1 |
| ZCCHC11 | mRNA | NDN | mRNA | mRNA-mRNA | 0.999969 | 1 |
| CHRFAM7A | mRNA | ADRB2 | mRNA | mRNA-mRNA | 0.999969 | 1 |
| RAB38 | mRNA | IGFBP3 | mRNA | mRNA-mRNA | 0.999969 | 1 |
| ADRB2 | mRNA | ADAMTS6 | mRNA | mRNA-mRNA | 0.999969 | 1 |
| SLC18B1 | mRNA | SGCE | mRNA | mRNA-mRNA | 0.999969 | 1 |
| PSMB8 | mRNA | GDA | mRNA | mRNA-mRNA | 0.999969 | 1 |
| LY6K | mRNA | LAMC3 | mRNA | mRNA-mRNA | 0.999969 | 1 |
| NCOA3 | mRNA | CXXC5 | mRNA | mRNA-mRNA | 0.999969 | 1 |
| RET | mRNA | PFDN4 | mRNA | mRNA-mRNA | 0.999968 | 1 |
| TMEM200A | mRNA | GPC5 | mRNA | mRNA-mRNA | 0.999968 | 1 |
| PSMB8 | mRNA | LY6K | mRNA | mRNA-mRNA | 0.999968 | 1 |
| WDR66 | mRNA | IQCJ-SCHIP1 | mRNA | mRNA-mRNA | 0.999968 | 1 |
| MCAM | mRNA | LAYN | mRNA | mRNA-mRNA | 0.999968 | 1 |
| NDN | mRNA | MLC1 | mRNA | mRNA-mRNA | 0.999968 | 1 |
| MSRB3 | mRNA | GSDMD | mRNA | mRNA-mRNA | 0.999967 | 1 |
| ZNF215 | mRNA | GDA | mRNA | mRNA-mRNA | 0.999967 | 1 |
| RPS6KB1 | mRNA | AGR3 | mRNA | mRNA-mRNA | 0.999967 | 1 |
| PDPN | mRNA | IGFBP3 | mRNA | mRNA-mRNA | 0.999967 | 1 |
| KRTAP2-3 | mRNA | CD274 | mRNA | mRNA-mRNA | 0.999967 | 1 |
| GRHL2 | mRNA | ABHD11 | mRNA | mRNA-mRNA | 0.999967 | 1 |
| GPC5 | mRNA | ADAMTS6 | mRNA | mRNA-mRNA | 0.999967 | 1 |
| PSMB8 | mRNA | CALD1 | mRNA | mRNA-mRNA | 0.999967 | 1 |
| SH3RF3 | mRNA | LRCH2 | mRNA | mRNA-mRNA | 0.999967 | 1 |
| SRPX | mRNA | CALD1 | mRNA | mRNA-mRNA | 0.999967 | 1 |
| SGCE | mRNA | MAEA | mRNA | mRNA-mRNA | 0.999966 | 1 |
| SAMD3 | mRNA | ABCB1 | mRNA | mRNA-mRNA | 0.999966 | 1 |
| TMEM200B | mRNA | CHRFAM7A | mRNA | mRNA-mRNA | 0.999966 | 1 |
| LAMC2 | mRNA | LAMA1 | mRNA | mRNA-mRNA | 0.999966 | 1 |
| LY6K | mRNA | LAYN | mRNA | mRNA-mRNA | 0.999966 | 1 |
| UCHL1 | mRNA | LAMC3 | mRNA | mRNA-mRNA | 0.999966 | 1 |
| FOXG1 | mRNA | CDC42EP3 | mRNA | mRNA-mRNA | 0.999966 | 1 |
| GPC5 | mRNA | CHRFAM7A | mRNA | mRNA-mRNA | 0.999966 | 1 |
| RHOJ | mRNA | CDC42EP3 | mRNA | mRNA-mRNA | 0.999966 | 1 |
| TMEM45B | mRNA | KRT80 | mRNA | mRNA-mRNA | 0.999965 | 1 |
| TMEM200A | mRNA | TMEM106A | mRNA | mRNA-mRNA | 0.999965 | 1 |
| RNF145 | mRNA | MYL9 | mRNA | mRNA-mRNA | 0.999965 | 1 |
| PFDN4 | mRNA | GFRA1 | mRNA | mRNA-mRNA | 0.999965 | 1 |
| TMEM200B | mRNA | ADRB2 | mRNA | mRNA-mRNA | 0.999965 | 1 |
| MICU3 | mRNA | FABP5 | mRNA | mRNA-mRNA | 0.999965 | 1 |
| RAB38 | mRNA | CST6 | mRNA | mRNA-mRNA | 0.999965 | 1 |
| PSG6 | mRNA | DPH3 | mRNA | mRNA-mRNA | 0.999965 | 1 |
| TMEM45B | mRNA | MALL | mRNA | mRNA-mRNA | 0.999965 | 1 |
| RHOJ | mRNA | ANKLE1 | mRNA | mRNA-mRNA | 0.999965 | 1 |
| ORC5 | mRNA | IDH1 | mRNA | mRNA-mRNA | 0.999965 | 1 |
| CXCL12 | mRNA | CDH1 | mRNA | mRNA-mRNA | 0.999965 | 1 |
| STEAP1B | mRNA | GDA | mRNA | mRNA-mRNA | 0.999965 | 1 |
| KRTAP2-3 | mRNA | KCNJ18 | mRNA | mRNA-mRNA | 0.999965 | 1 |
| ZNF22 | mRNA | FOSL1 | mRNA | mRNA-mRNA | 0.999964 | 1 |
| STEAP1B | mRNA | ETV5 | mRNA | mRNA-mRNA | 0.999964 | 1 |
| SLC18B1 | mRNA | MAEA | mRNA | mRNA-mRNA | 0.999964 | 1 |
| UCHL1 | mRNA | NDN | mRNA | mRNA-mRNA | 0.999964 | 1 |
| PDPN | mRNA | DGLUCY | mRNA | mRNA-mRNA | 0.999964 | 1 |
| IGFBP3 | mRNA | CST6 | mRNA | mRNA-mRNA | 0.999964 | 1 |
| DOLK | mRNA | C5AR2 | mRNA | mRNA-mRNA | 0.999964 | 1 |
| SPAG16 | mRNA | LAYN | mRNA | mRNA-mRNA | 0.999964 | 1 |
| TMEM200B | mRNA | MCAM | mRNA | mRNA-mRNA | 0.999964 | 1 |
| GAREM2 | mRNA | FGD1 | mRNA | mRNA-mRNA | 0.999964 | 1 |
| TRIM47 | mRNA | MAEA | mRNA | mRNA-mRNA | 0.999964 | 1 |
| MICU3 | mRNA | LAYN | mRNA | mRNA-mRNA | 0.999964 | 1 |
| SH3RF3 | mRNA | CST6 | mRNA | mRNA-mRNA | 0.999963 | 1 |
| SNURF | mRNA | LAYN | mRNA | mRNA-mRNA | 0.999963 | 1 |
| ORC5 | mRNA | AQP3 | mRNA | mRNA-mRNA | 0.999963 | 1 |
| SPAG16 | mRNA | FABP5 | mRNA | mRNA-mRNA | 0.999963 | 1 |
| SRPX | mRNA | RAB3B | mRNA | mRNA-mRNA | 0.999963 | 1 |
| SRPX | mRNA | MLC1 | mRNA | mRNA-mRNA | 0.999963 | 1 |
| SNRPN | mRNA | LY6K | mRNA | mRNA-mRNA | 0.999963 | 1 |
| EXT1 | mRNA | ANXA3 | mRNA | mRNA-mRNA | 0.999963 | 1 |
| ZCCHC11 | mRNA | SLC18B1 | mRNA | mRNA-mRNA | 0.999963 | 1 |
| PROS1 | mRNA | AJUBA | mRNA | mRNA-mRNA | 0.999963 | 1 |
| SNURF | mRNA | SNRPN | mRNA | mRNA-mRNA | 0.999963 | 1 |
| RFLNB | mRNA | ELAVL2 | mRNA | mRNA-mRNA | 0.999963 | 1 |
| LOX | mRNA | FGD1 | mRNA | mRNA-mRNA | 0.999963 | 1 |
| TRIM47 | mRNA | SGCE | mRNA | mRNA-mRNA | 0.999963 | 1 |
| ZNF215 | mRNA | UCHL1 | mRNA | mRNA-mRNA | 0.999962 | 1 |
| RNF217 | mRNA | OSBPL3 | mRNA | mRNA-mRNA | 0.999962 | 1 |
| NETO2 | mRNA | COTL1 | mRNA | mRNA-mRNA | 0.999962 | 1 |
| NETO2 | mRNA | CDIP1 | mRNA | mRNA-mRNA | 0.999962 | 1 |
| TMEM200A | mRNA | PSG8 | mRNA | mRNA-mRNA | 0.999962 | 1 |
| GNB2 | mRNA | FGFR2 | mRNA | mRNA-mRNA | 0.999962 | 1 |
| ZNF717 | mRNA | ANXA6 | mRNA | mRNA-mRNA | 0.999962 | 1 |
| POF1B | mRNA | NCOA3 | mRNA | mRNA-mRNA | 0.999962 | 1 |
| UCHL1 | mRNA | ADAMTS6 | mRNA | mRNA-mRNA | 0.999961 | 1 |
| UCHL1 | mRNA | TMEM200A | mRNA | mRNA-mRNA | 0.999961 | 1 |
| MAEA | mRNA | FGFBP1 | mRNA | mRNA-mRNA | 0.999961 | 1 |
| KCNJ18 | mRNA | CD274 | mRNA | mRNA-mRNA | 0.999961 | 1 |
| TBX18 | mRNA | SNURF | mRNA | mRNA-mRNA | 0.999961 | 1 |
| CDH1 | mRNA | CA12 | mRNA | mRNA-mRNA | 0.999961 | 1 |
| KRTAP2-3 | mRNA | FGF5 | mRNA | mRNA-mRNA | 0.999961 | 1 |
| TMEM200A | mRNA | MLC1 | mRNA | mRNA-mRNA | 0.999961 | 1 |
| RNF217 | mRNA | MICU3 | mRNA | mRNA-mRNA | 0.999961 | 1 |
| RAMP2 | mRNA | CDC42EP3 | mRNA | mRNA-mRNA | 0.999960 | 1 |
| ZCCHC11 | mRNA | SRPX | mRNA | mRNA-mRNA | 0.999960 | 1 |
| TBX18 | mRNA | LY6K | mRNA | mRNA-mRNA | 0.999960 | 1 |
| SLC18B1 | mRNA | KRTAP2-3 | mRNA | mRNA-mRNA | 0.999960 | 1 |
| SGCE | mRNA | KCNJ18 | mRNA | mRNA-mRNA | 0.999960 | 1 |
| MLC1 | mRNA | ANKLE1 | mRNA | mRNA-mRNA | 0.999960 | 1 |
| NT5E | mRNA | LHFPL6 | mRNA | mRNA-mRNA | 0.999960 | 1 |
| EXT1 | mRNA | ANXA1 | mRNA | mRNA-mRNA | 0.999960 | 1 |
| VIM | mRNA | PPP1R18 | mRNA | mRNA-mRNA | 0.999959 | 1 |
| UCHL1 | mRNA | PSMB8 | mRNA | mRNA-mRNA | 0.999959 | 1 |
| UCHL1 | mRNA | MSN | mRNA | mRNA-mRNA | 0.999959 | 1 |
| ZNF215 | mRNA | ADRB2 | mRNA | mRNA-mRNA | 0.999959 | 1 |
| IL6 | mRNA | ABCB1 | mRNA | mRNA-mRNA | 0.999959 | 1 |
| MYL9 | mRNA | ADGRL2 | mRNA | mRNA-mRNA | 0.999959 | 1 |
| TMEM106A | mRNA | CHRFAM7A | mRNA | mRNA-mRNA | 0.999959 | 1 |
| RNF217 | mRNA | FABP5 | mRNA | mRNA-mRNA | 0.999958 | 1 |
| NNMT | mRNA | LAMC3 | mRNA | mRNA-mRNA | 0.999958 | 1 |
| RYR1 | mRNA | CTNNAL1 | mRNA | mRNA-mRNA | 0.999958 | 1 |
| RBFOX3 | mRNA | ETV5 | mRNA | mRNA-mRNA | 0.999958 | 1 |
| UCHL1 | mRNA | STEAP1B | mRNA | mRNA-mRNA | 0.999958 | 1 |
| SH3RF3 | mRNA | DGLUCY | mRNA | mRNA-mRNA | 0.999958 | 1 |
| ZNF717 | mRNA | TSPO | mRNA | mRNA-mRNA | 0.999958 | 1 |
| SAMD3 | mRNA | PDPN | mRNA | mRNA-mRNA | 0.999957 | 1 |
| UCHL1 | mRNA | MCAM | mRNA | mRNA-mRNA | 0.999957 | 1 |
| RHOJ | mRNA | KCNJ18 | mRNA | mRNA-mRNA | 0.999957 | 1 |
| SPAG16 | mRNA | LRCH2 | mRNA | mRNA-mRNA | 0.999957 | 1 |
| ST6GALNAC3 | mRNA | SNRPN | mRNA | mRNA-mRNA | 0.999957 | 1 |
| RAB3B | mRNA | NDN | mRNA | mRNA-mRNA | 0.999957 | 1 |
| FABP5 | mRNA | B4GALT5 | mRNA | mRNA-mRNA | 0.999957 | 1 |
| TMEM200B | mRNA | TMEM200A | mRNA | mRNA-mRNA | 0.999957 | 1 |
| FGFBP1 | mRNA | CDC42EP3 | mRNA | mRNA-mRNA | 0.999957 | 1 |
| SH3PXD2A | mRNA | CD109 | mRNA | mRNA-mRNA | 0.999957 | 1 |
| RNF145 | mRNA | IL1A | mRNA | mRNA-mRNA | 0.999957 | 1 |
| SRPX | mRNA | GDA | mRNA | mRNA-mRNA | 0.999957 | 1 |
| RNF217 | mRNA | B4GALT5 | mRNA | mRNA-mRNA | 0.999956 | 1 |
| ST6GALNAC3 | mRNA | GSDMD | mRNA | mRNA-mRNA | 0.999956 | 1 |
| MLC1 | mRNA | CDC42EP3 | mRNA | mRNA-mRNA | 0.999956 | 1 |
| RHOJ | mRNA | FMN2 | mRNA | mRNA-mRNA | 0.999956 | 1 |
| GSDMD | mRNA | FOSL1 | mRNA | mRNA-mRNA | 0.999956 | 1 |
| RTN1 | mRNA | PROS1 | mRNA | mRNA-mRNA | 0.999956 | 1 |
| ELAVL2 | mRNA | APOBEC3G | mRNA | mRNA-mRNA | 0.999956 | 1 |
| LY6K | mRNA | IGFBP3 | mRNA | mRNA-mRNA | 0.999956 | 1 |
| ST6GALNAC3 | mRNA | RSPO4 | mRNA | mRNA-mRNA | 0.999956 | 1 |
| LIMA1 | mRNA | ANKRD1 | mRNA | mRNA-mRNA | 0.999955 | 1 |
| CD274 | mRNA | ARHGAP40 | mRNA | mRNA-mRNA | 0.999955 | 1 |
| SH3KBP1 | mRNA | PPP1R14C | mRNA | mRNA-mRNA | 0.999955 | 1 |
| TPM2 | mRNA | SNURF | mRNA | mRNA-mRNA | 0.999955 | 1 |
| RAB3B | mRNA | PSMB8 | mRNA | mRNA-mRNA | 0.999955 | 1 |
| VIM | mRNA | MMP1 | mRNA | mRNA-mRNA | 0.999955 | 1 |
| PRR15L | mRNA | ACKR3 | mRNA | mRNA-mRNA | 0.999955 | 1 |
| ZNF215 | mRNA | CHRFAM7A | mRNA | mRNA-mRNA | 0.999955 | 1 |
| TMEM106A | mRNA | ARHGAP40 | mRNA | mRNA-mRNA | 0.999955 | 1 |
| RHOJ | mRNA | FGFBP1 | mRNA | mRNA-mRNA | 0.999955 | 1 |
| SV2A | mRNA | BEX1 | mRNA | mRNA-mRNA | 0.999955 | 1 |
| SLC18B1 | mRNA | MLC1 | mRNA | mRNA-mRNA | 0.999955 | 1 |
| CALCOCO1 | mRNA | ANKRD1 | mRNA | mRNA-mRNA | 0.999955 | 1 |
| STEAP1B | mRNA | PSMB8 | mRNA | mRNA-mRNA | 0.999955 | 1 |
| KRTAP2-3 | mRNA | FGFBP1 | mRNA | mRNA-mRNA | 0.999955 | 1 |
| LIMA1 | mRNA | ANXA6 | mRNA | mRNA-mRNA | 0.999954 | 1 |
| PSG6 | mRNA | AJUBA | mRNA | mRNA-mRNA | 0.999954 | 1 |
| ZNF215 | mRNA | LAMC3 | mRNA | mRNA-mRNA | 0.999954 | 1 |
| LAMC2 | mRNA | IGFBP3 | mRNA | mRNA-mRNA | 0.999954 | 1 |
| ST6GALNAC3 | mRNA | CSPG4 | mRNA | mRNA-mRNA | 0.999954 | 1 |
| TMEM106A | mRNA | SRPX | mRNA | mRNA-mRNA | 0.999954 | 1 |
| S1PR1 | mRNA | MLC1 | mRNA | mRNA-mRNA | 0.999954 | 1 |
| TMEM106A | mRNA | OR2W3 | mRNA | mRNA-mRNA | 0.999954 | 1 |
| IL18 | mRNA | GPX1 | mRNA | mRNA-mRNA | 0.999954 | 1 |
| UCHL1 | mRNA | ST6GALNAC3 | mRNA | mRNA-mRNA | 0.999954 | 1 |
| TRIM47 | mRNA | PLAG1 | mRNA | mRNA-mRNA | 0.999954 | 1 |
| ZNF215 | mRNA | NDN | mRNA | mRNA-mRNA | 0.999953 | 1 |
| NNMT | mRNA | MCAM | mRNA | mRNA-mRNA | 0.999953 | 1 |
| MYOM3 | mRNA | HEG1 | mRNA | mRNA-mRNA | 0.999953 | 1 |
| FERMT1 | mRNA | FBLN5 | mRNA | mRNA-mRNA | 0.999953 | 1 |
| LSP1 | mRNA | DPH3 | mRNA | mRNA-mRNA | 0.999953 | 1 |
| UCHL1 | mRNA | RAB3B | mRNA | mRNA-mRNA | 0.999953 | 1 |
| TMEM200B | mRNA | MSN | mRNA | mRNA-mRNA | 0.999953 | 1 |
| RASIP1 | mRNA | BTG3 | mRNA | mRNA-mRNA | 0.999953 | 1 |
| AQP3 | mRNA | AGR2 | mRNA | mRNA-mRNA | 0.999953 | 1 |
| PSMB8 | mRNA | IGFBP3 | mRNA | mRNA-mRNA | 0.999953 | 1 |
| C5AR2 | mRNA | BSPRY | mRNA | mRNA-mRNA | 0.999953 | 1 |
| LAMC3 | mRNA | GPC5 | mRNA | mRNA-mRNA | 0.999953 | 1 |
| NNMT | mRNA | MSN | mRNA | mRNA-mRNA | 0.999953 | 1 |
| SAMD3 | mRNA | IL6 | mRNA | mRNA-mRNA | 0.999952 | 1 |
| WDR66 | mRNA | ITGA6 | mRNA | mRNA-mRNA | 0.999952 | 1 |
| ZFPM2 | mRNA | OSBPL3 | mRNA | mRNA-mRNA | 0.999952 | 1 |
| UAP1 | mRNA | ITGA4 | mRNA | mRNA-mRNA | 0.999952 | 1 |
| TMEM106A | mRNA | GDA | mRNA | mRNA-mRNA | 0.999952 | 1 |
| ZNF215 | mRNA | RAB3B | mRNA | mRNA-mRNA | 0.999952 | 1 |
| PSG8 | mRNA | ETV5 | mRNA | mRNA-mRNA | 0.999952 | 1 |
| LRCH2 | mRNA | CNN1 | mRNA | mRNA-mRNA | 0.999952 | 1 |
| SH3RF3 | mRNA | SAMD3 | mRNA | mRNA-mRNA | 0.999951 | 1 |
| ZNF215 | mRNA | SRPX | mRNA | mRNA-mRNA | 0.999951 | 1 |
| TMEM200B | mRNA | GSDMD | mRNA | mRNA-mRNA | 0.999951 | 1 |
| LRCH2 | mRNA | LONRF3 | mRNA | mRNA-mRNA | 0.999951 | 1 |
| TMEM200B | mRNA | RAB3B | mRNA | mRNA-mRNA | 0.999951 | 1 |
| TMEM200B | mRNA | PSMB8 | mRNA | mRNA-mRNA | 0.999951 | 1 |
| RFLNB | mRNA | CT45A5 | mRNA | mRNA-mRNA | 0.999951 | 1 |
| TSTD1 | mRNA | BSPRY | mRNA | mRNA-mRNA | 0.999951 | 1 |
| ZCCHC11 | mRNA | ANKLE1 | mRNA | mRNA-mRNA | 0.999951 | 1 |
| TMEM106A | mRNA | ADRB2 | mRNA | mRNA-mRNA | 0.999951 | 1 |
| TMEM106A | mRNA | S1PR1 | mRNA | mRNA-mRNA | 0.999951 | 1 |
| FGF5 | mRNA | CDC42EP3 | mRNA | mRNA-mRNA | 0.999951 | 1 |
| SNURF | mRNA | MCAM | mRNA | mRNA-mRNA | 0.999951 | 1 |
| LAYN | mRNA | DGLUCY | mRNA | mRNA-mRNA | 0.999951 | 1 |
| SNURF | mRNA | MSN | mRNA | mRNA-mRNA | 0.999951 | 1 |
| PLAG1 | mRNA | KCNJ18 | mRNA | mRNA-mRNA | 0.999951 | 1 |
| SRPX | mRNA | EVC | mRNA | mRNA-mRNA | 0.999951 | 1 |
| FOXG1 | mRNA | CD109 | mRNA | mRNA-mRNA | 0.999950 | 1 |
| STEAP1B | mRNA | CALD1 | mRNA | mRNA-mRNA | 0.999950 | 1 |
| TMEM200A | mRNA | OR2W3 | mRNA | mRNA-mRNA | 0.999950 | 1 |
| TBX18 | mRNA | ST6GALNAC3 | mRNA | mRNA-mRNA | 0.999950 | 1 |
| LOX | mRNA | GAREM2 | mRNA | mRNA-mRNA | 0.999950 | 1 |
| TMEM45B | mRNA | RPS6KB1 | mRNA | mRNA-mRNA | 0.999950 | 1 |
| PSMB8 | mRNA | LAMC3 | mRNA | mRNA-mRNA | 0.999950 | 1 |
| DPH3 | mRNA | AJUBA | mRNA | mRNA-mRNA | 0.999950 | 1 |
| MLC1 | mRNA | CHRFAM7A | mRNA | mRNA-mRNA | 0.999950 | 1 |
| TMEM200B | mRNA | NDN | mRNA | mRNA-mRNA | 0.999950 | 1 |
| PTX3 | mRNA | FXYD5 | mRNA | mRNA-mRNA | 0.999950 | 1 |
| TSTD1 | mRNA | TP53I11 | mRNA | mRNA-mRNA | 0.999950 | 1 |
| EXT1 | mRNA | CNN1 | mRNA | mRNA-mRNA | 0.999949 | 1 |
| ELAVL2 | mRNA | DZIP1 | mRNA | mRNA-mRNA | 0.999949 | 1 |
| MALL | mRNA | KRT80 | mRNA | mRNA-mRNA | 0.999949 | 1 |
| TMEM200A | mRNA | S1PR1 | mRNA | mRNA-mRNA | 0.999949 | 1 |
| MLC1 | mRNA | MAEA | mRNA | mRNA-mRNA | 0.999949 | 1 |
| FGD1 | mRNA | ETV5 | mRNA | mRNA-mRNA | 0.999949 | 1 |
| STARD10 | mRNA | ESRP1 | mRNA | mRNA-mRNA | 0.999949 | 1 |
| SGCE | mRNA | NT5E | mRNA | mRNA-mRNA | 0.999949 | 1 |
| CLDN7 | mRNA | ABHD11 | mRNA | mRNA-mRNA | 0.999949 | 1 |
| PGM1 | mRNA | CLIC4 | mRNA | mRNA-mRNA | 0.999949 | 1 |
| ZCCHC11 | mRNA | OR2W3 | mRNA | mRNA-mRNA | 0.999949 | 1 |
| RNF217 | mRNA | FGD1 | mRNA | mRNA-mRNA | 0.999949 | 1 |
| ZCCHC11 | mRNA | PSG8 | mRNA | mRNA-mRNA | 0.999948 | 1 |
| RNF152 | mRNA | GRHL2 | mRNA | mRNA-mRNA | 0.999948 | 1 |
| ZCCHC11 | mRNA | CHRFAM7A | mRNA | mRNA-mRNA | 0.999948 | 1 |
| SGCE | mRNA | FERMT1 | mRNA | mRNA-mRNA | 0.999948 | 1 |
| MSRB3 | mRNA | MSN | mRNA | mRNA-mRNA | 0.999948 | 1 |
| SV2A | mRNA | PLAG1 | mRNA | mRNA-mRNA | 0.999948 | 1 |
| SYTL2 | mRNA | SFXN2 | mRNA | mRNA-mRNA | 0.999948 | 1 |
| UCHL1 | mRNA | PSG8 | mRNA | mRNA-mRNA | 0.999948 | 1 |
| RAB3B | mRNA | PSG8 | mRNA | mRNA-mRNA | 0.999948 | 1 |
| TMEM106A | mRNA | CALD1 | mRNA | mRNA-mRNA | 0.999948 | 1 |
| FOXG1 | mRNA | BSPRY | mRNA | mRNA-mRNA | -0.999948 | -1 |
| THSD1 | mRNA | ACKR3 | mRNA | mRNA-mRNA | -0.999949 | -1 |
| MFSD1 | mRNA | CSPG4 | mRNA | mRNA-mRNA | -0.999949 | -1 |
| MLC1 | mRNA | BATF | mRNA | mRNA-mRNA | -0.999949 | -1 |
| ZNF215 | mRNA | CLDN7 | mRNA | mRNA-mRNA | -0.999949 | -1 |
| LYPD6B | mRNA | CLIC4 | mRNA | mRNA-mRNA | -0.999949 | -1 |
| FMN2 | mRNA | ADIRF | mRNA | mRNA-mRNA | -0.999949 | -1 |
| PLEKHO2 | mRNA | MFSD1 | mRNA | mRNA-mRNA | -0.999950 | -1 |
| LRRN4 | mRNA | BSCL2 | mRNA | mRNA-mRNA | -0.999950 | -1 |
| GRHL2 | mRNA | GPC5 | mRNA | mRNA-mRNA | -0.999950 | -1 |
| SPEM1 | mRNA | ARMT1 | mRNA | mRNA-mRNA | -0.999950 | -1 |
| SLC18B1 | mRNA | BATF | mRNA | mRNA-mRNA | -0.999950 | -1 |
| SV2A | mRNA | DDR1 | mRNA | mRNA-mRNA | -0.999951 | -1 |
| TP53I11 | mRNA | S1PR1 | mRNA | mRNA-mRNA | -0.999951 | -1 |
| ZNF597 | mRNA | NTNG1 | mRNA | mRNA-mRNA | -0.999952 | -1 |
| SLC7A2 | mRNA | G0S2 | mRNA | mRNA-mRNA | -0.999952 | -1 |
| SLC7A2 | mRNA | ARHGAP40 | mRNA | mRNA-mRNA | -0.999952 | -1 |
| PGM1 | mRNA | LYPD6B | mRNA | mRNA-mRNA | -0.999952 | -1 |
| MFSD1 | mRNA | FOSL1 | mRNA | mRNA-mRNA | -0.999952 | -1 |
| TSTD1 | mRNA | NDN | mRNA | mRNA-mRNA | -0.999953 | -1 |
| SGCE | mRNA | DOLK | mRNA | mRNA-mRNA | -0.999953 | -1 |
| TP53I11 | mRNA | PSG8 | mRNA | mRNA-mRNA | -0.999953 | -1 |
| OR2W3 | mRNA | GPD2 | mRNA | mRNA-mRNA | -0.999954 | -1 |
| BEX1 | mRNA | ADIRF | mRNA | mRNA-mRNA | -0.999954 | -1 |
| GNB2 | mRNA | FERMT1 | mRNA | mRNA-mRNA | -0.999954 | -1 |
| SFXN2 | mRNA | HMGA1P8 | mRNA | mRNA-mRNA | -0.999954 | -1 |
| TMTC1 | mRNA | RNF145 | mRNA | mRNA-mRNA | -0.999954 | -1 |
| IGFBP3 | mRNA | ABCC3 | mRNA | mRNA-mRNA | -0.999954 | -1 |
| NDN | mRNA | BSPRY | mRNA | mRNA-mRNA | -0.999955 | -1 |
| RNF145 | mRNA | KYNU | mRNA | mRNA-mRNA | -0.999956 | -1 |
| THSD1 | mRNA | PRR15L | mRNA | mRNA-mRNA | -0.999956 | -1 |
| PRLR | mRNA | NNMT | mRNA | mRNA-mRNA | -0.999957 | -1 |
| MLC1 | mRNA | BSPRY | mRNA | mRNA-mRNA | -0.999957 | -1 |
| PPP1R14C | mRNA | ABCC3 | mRNA | mRNA-mRNA | -0.999957 | -1 |
| FMN2 | mRNA | ESRP2 | mRNA | mRNA-mRNA | -0.999957 | -1 |
| HIPK1 | mRNA | FMN2 | mRNA | mRNA-mRNA | -0.999958 | -1 |
| SNURF | mRNA | PRLR | mRNA | mRNA-mRNA | -0.999958 | -1 |
| PRLR | mRNA | LAMC3 | mRNA | mRNA-mRNA | -0.999959 | -1 |
| SPAG16 | mRNA | PRR15L | mRNA | mRNA-mRNA | -0.999959 | -1 |
| ZNF215 | mRNA | ABHD11 | mRNA | mRNA-mRNA | -0.999959 | -1 |
| ZCCHC11 | mRNA | BATF | mRNA | mRNA-mRNA | -0.999961 | -1 |
| OR2W3 | mRNA | BATF | mRNA | mRNA-mRNA | -0.999961 | -1 |
| SYTL2 | mRNA | HMGA1P8 | mRNA | mRNA-mRNA | -0.999961 | -1 |
| GRHL2 | mRNA | ADAMTS6 | mRNA | mRNA-mRNA | -0.999961 | -1 |
| TRIM16L | mRNA | SH3GLB1 | mRNA | mRNA-mRNA | -0.999962 | -1 |
| PPM1D | mRNA | MLLT11 | mRNA | mRNA-mRNA | -0.999962 | -1 |
| POF1B | mRNA | HMGA1P8 | mRNA | mRNA-mRNA | -0.999963 | -1 |
| TP53I11 | mRNA | OR2W3 | mRNA | mRNA-mRNA | -0.999963 | -1 |
| RBFOX3 | mRNA | PRR15L | mRNA | mRNA-mRNA | -0.999964 | -1 |
| MYL9 | mRNA | GFRA1 | mRNA | mRNA-mRNA | -0.999964 | -1 |
| PPM1D | mRNA | MXRA7 | mRNA | mRNA-mRNA | -0.999965 | -1 |
| FGFR2 | mRNA | FBLN5 | mRNA | mRNA-mRNA | -0.999965 | -1 |
| TP53I11 | mRNA | ADRB2 | mRNA | mRNA-mRNA | -0.999966 | -1 |
| SNRPN | mRNA | PRLR | mRNA | mRNA-mRNA | -0.999966 | -1 |
| TP53I11 | mRNA | MLC1 | mRNA | mRNA-mRNA | -0.999966 | -1 |
| PSMB8 | mRNA | PRLR | mRNA | mRNA-mRNA | -0.999967 | -1 |
| IL1A | mRNA | GFRA1 | mRNA | mRNA-mRNA | -0.999967 | -1 |
| PRR15L | mRNA | FGD1 | mRNA | mRNA-mRNA | -0.999967 | -1 |
| TP53I11 | mRNA | GDA | mRNA | mRNA-mRNA | -0.999967 | -1 |
| RFTN1 | mRNA | CDH1 | mRNA | mRNA-mRNA | -0.999967 | -1 |
| ZCCHC11 | mRNA | TP53I11 | mRNA | mRNA-mRNA | -0.999967 | -1 |
| GFRA1 | mRNA | ADGRL2 | mRNA | mRNA-mRNA | -0.999968 | -1 |
| TPD52L1 | mRNA | GOLGA8A | mRNA | mRNA-mRNA | -0.999969 | -1 |
| LONRF3 | mRNA | ABCC3 | mRNA | mRNA-mRNA | -0.999969 | -1 |
| ZFPM2 | mRNA | SLC7A2 | mRNA | mRNA-mRNA | -0.999970 | -1 |
| TP53I11 | mRNA | CALD1 | mRNA | mRNA-mRNA | -0.999971 | -1 |
| RFTN1 | mRNA | CA12 | mRNA | mRNA-mRNA | -0.999971 | -1 |
| NT5E | mRNA | FAM160B1 | mRNA | mRNA-mRNA | -0.999971 | -1 |
| TSTD1 | mRNA | CHRFAM7A | mRNA | mRNA-mRNA | -0.999971 | -1 |
| VIM | mRNA | SLC39A6 | mRNA | mRNA-mRNA | -0.999971 | -1 |
| PRLR | mRNA | IGFBP3 | mRNA | mRNA-mRNA | -0.999972 | -1 |
| TP53I11 | mRNA | ADAMTS6 | mRNA | mRNA-mRNA | -0.999972 | -1 |
| TBX18 | mRNA | PRLR | mRNA | mRNA-mRNA | -0.999972 | -1 |
| SPTSSB | mRNA | SPDL1 | mRNA | mRNA-mRNA | -0.999972 | -1 |
| S1PR1 | mRNA | BSPRY | mRNA | mRNA-mRNA | -0.999973 | -1 |
| HMGA1P8 | mRNA | CXXC5 | mRNA | mRNA-mRNA | -0.999973 | -1 |
| PRLR | mRNA | MCAM | mRNA | mRNA-mRNA | -0.999974 | -1 |
| PHYHD1 | mRNA | IRX2 | mRNA | mRNA-mRNA | -0.999974 | -1 |
| TP53I11 | mRNA | NDN | mRNA | mRNA-mRNA | -0.999974 | -1 |
| HLA-A | mRNA | EMC10 | mRNA | mRNA-mRNA | -0.999975 | -1 |
| ZNF597 | mRNA | HEG1 | mRNA | mRNA-mRNA | -0.999975 | -1 |
| RPS6KB1 | mRNA | RFTN1 | mRNA | mRNA-mRNA | -0.999976 | -1 |
| PFDN4 | mRNA | CMTM3 | mRNA | mRNA-mRNA | -0.999976 | -1 |
| PDK3 | mRNA | HLA-DPB1 | mRNA | mRNA-mRNA | -0.999977 | -1 |
| CD274 | mRNA | BATF | mRNA | mRNA-mRNA | -0.999977 | -1 |
| TMEM106A | mRNA | BATF | mRNA | mRNA-mRNA | -0.999978 | -1 |
| PRR15L | mRNA | ETV5 | mRNA | mRNA-mRNA | -0.999978 | -1 |
| PSG8 | mRNA | BATF | mRNA | mRNA-mRNA | -0.999978 | -1 |
| THSD1 | mRNA | ABCC3 | mRNA | mRNA-mRNA | -0.999978 | -1 |
| PRLR | mRNA | LY6K | mRNA | mRNA-mRNA | -0.999978 | -1 |
| TSTD1 | mRNA | SRPX | mRNA | mRNA-mRNA | -0.999979 | -1 |
| PRLR | mRNA | LAYN | mRNA | mRNA-mRNA | -0.999980 | -1 |
| TP53I11 | mRNA | TMEM106A | mRNA | mRNA-mRNA | -0.999980 | -1 |
| ESRP2 | mRNA | BEX1 | mRNA | mRNA-mRNA | -0.999982 | -1 |
| TP53I11 | mRNA | SRPX | mRNA | mRNA-mRNA | -0.999983 | -1 |
| SH3PXD2A | mRNA | BSCL2 | mRNA | mRNA-mRNA | -0.999983 | -1 |
| TP53I11 | mRNA | RAB3B | mRNA | mRNA-mRNA | -0.999983 | -1 |
| TSTD1 | mRNA | S1PR1 | mRNA | mRNA-mRNA | -0.999984 | -1 |
| RBFOX3 | mRNA | ACKR3 | mRNA | mRNA-mRNA | -0.999984 | -1 |
| PLAG1 | mRNA | DOLK | mRNA | mRNA-mRNA | -0.999984 | -1 |
| ZNF215 | mRNA | GRHL2 | mRNA | mRNA-mRNA | -0.999985 | -1 |
| TP53I11 | mRNA | TMEM200A | mRNA | mRNA-mRNA | -0.999985 | -1 |
| SLC39A6 | mRNA | PPP1R18 | mRNA | mRNA-mRNA | -0.999986 | -1 |
| TP53I11 | mRNA | CHRFAM7A | mRNA | mRNA-mRNA | -0.999988 | -1 |
| EMC10 | mRNA | CNN1 | mRNA | mRNA-mRNA | -0.999989 | -1 |
| PRLR | mRNA | GSDMD | mRNA | mRNA-mRNA | -0.999990 | -1 |
| PRLR | mRNA | MSN | mRNA | mRNA-mRNA | -0.999992 | -1 |
| SLC7A2 | mRNA | OSBPL3 | mRNA | mRNA-mRNA | -0.999994 | -1 |
| FGFR2 | mRNA | FERMT1 | mRNA | mRNA-mRNA | -0.999996 | -1 |
| DE-lncRNA=Differentially expressed lncRNAs;DE-mRNAs=Differentially expressed mRNAs | | | | | | |
| R value = Coefficient of correlation; | | | | | | |
| "Type= 1" equals to positive relationship; "Type= -1" equals to negative relationship. | | | | | | |
