# Supplementary material for: Integrative Analysis of the Doxorubicin-Associated LncRNA–mRNA Network Identifies Chemoresistance-Associated lnc-TRDMT1-5 as a Biomarker of Breast Cancer Progression
Source: Front Genet. 2020 May 29;11:566. doi: 10.3389/fgene.2020.00566 (PMC7272716; doi:10.3389/fgene.2020.00566)
Supplement: Supplementary file 2 [file Table_2.DOCX]

Supplementary Material

## Supplementary Figure S1


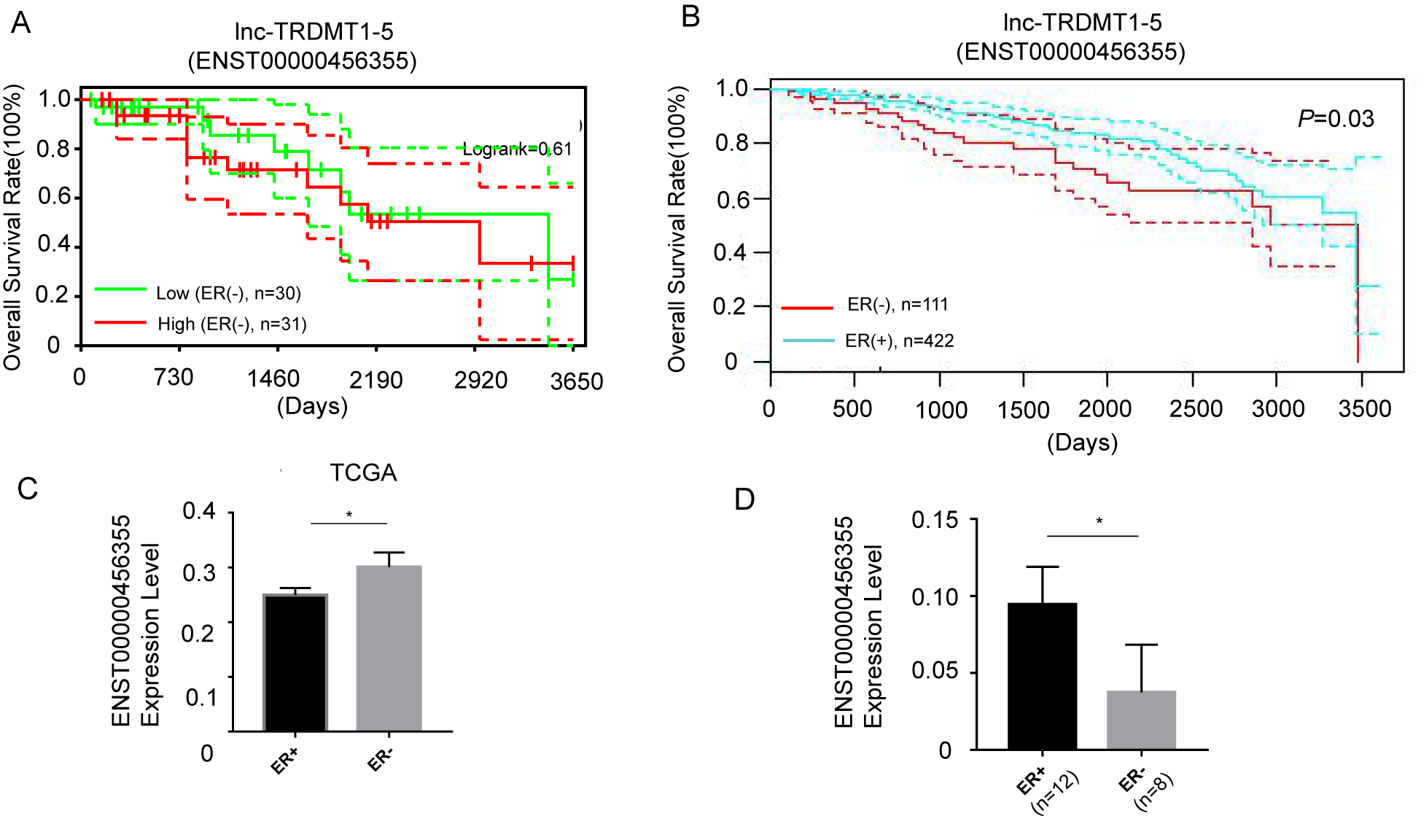


**Supplementary Figure S1.** (A) Overall survival curves of lnc-TRDMT1-5 expression level for ER-negative patients from TCGA database (n=61). Horizontal axis: overall survival time, days; vertical axis: survival rate. (B) The overall survival curves between total ER-positive patients (n=422) and ER-negative patients (n=111) in the lnc-TRDMT1-5 expression profile from TCGA database. (C) The expression level of lnc-TRDMT1-5 in ER-negative patients (n=122) and ER-positive patients (n=451) in TCGA database. (D) The expression level of lnc-TRDMT1-5 in ER-positive patients (n=12) and ER-negative patients (n=8). **P*<0.05.
